# Comparison of outcomes after SMILE using VISUMAX 800 and VISUMAX 500 for correcting myopia and myopic astigmatism: a systematic review and meta-analysis

**DOI:** 10.3389/fmed.2026.1899883

**Published:** 2026-10-07

**Authors:** Gang Li, Yinganqi Li

**Affiliations:** 1 Beijing Hospital of Integrated Traditional Chinese and Western Medicine, Affiliated Hospital of Integrated Traditional Chinese and Western Medicine, Beijing University of Chinese Medicine, Beijing, China; 2 Department of Statistics, University of Illinois Urbana-Champaign, Urbana, IL, United States

**Keywords:** keratorefractive lenticule extraction, meta-analysis, myopia, SMILE pro, VisuMax 800

## Abstract

**Background:**

The aim of this study was to compare the outcomes of correcting myopia and myopic astigmatism between small incision lenticule extraction (SMILE) surgery performed using VisuMax 500 (SMILE group) and that using VisuMax 800 (SMILE Pro group).

**Methods:**

PubMed, EMBASE, and the Cochrane Central Register of Controlled Trials were searched up to February 2026 to identify relevant studies. The PRISMA guidelines were followed. Primary outcomes were postoperative efficacy, safety, and predictability at the end of follow-up. Secondary outcomes were the postoperative high-order aberrations (HOAs) parameters and the vector analysis parameters.

**Results:**

A total of nine studies including 1,692 eyes were involved, with 1,036 eyes undergoing SMILE surgery, 656 eyes undergoing SMILE Pro surgery. All of the 9 selected studies were nonrandomized comparative studies (NRCSs) with the follow-up period ranged from 1 to 6 months. Compared with SMILE, SMILE Pro group had better stability of corrected vision (OR, 0.47; 95% CI, 0.25 to 0.86). As for cylinder and vector analysis, SMILE group showed a marginally greater residual cylinder than the SMILE Pro group (MD=−0.06; 95% CI, −0.13 to 0.00). There was statistically significant difference in |AE| between the two groups (MD=4.29; 95% CI, 2.34 to 6.24), suggesting that the SMILE group tended toward under-correction, while the SMILE Pro group tended toward over-correction. The post-operative UDVA, spherical equivalent (SE), and higher order aberrations and other vector analysis parameters between the two groups were not statistically significant.

**Conclusions:**

Within the limitations of this study, we did not detect evidence of statistical difference between SMILE and SMILE Pro surgical procedures when used for the treatment of myopia and astigmatism. SMILE Pro using VISUMAX 800 provided more accurate refractive cylinder outcomes. Further multicentre studies with larger sample sizes and longer follow-up are warranted to confirm VisuMax 800 system superior practical benefits in surgical workflow, refractive outcomes and patient comfort.

## 1 Introduction

Small incision lenticule extraction (SMILE) was introduced in 2011, characterized by using a femtosecond laser to create an accurate intrastromal lenticule, which is then removed through a small incision (1). The unique all-in-one procedure that uses only one femtosecond laser has made SMILE popular and has brought keratorefractive surgery into the era of Keratorefractive lenticule extraction (KLEx) (2).

Safety, effectiveness and optical quality have always been the goals of keratorefractive surgery. Compared with traditional laser in situ keratomileusis (LASIK) and photorefractive keratectomy (PRK), SMILE has shown good and satisfying outcomes and long-term clinical results (3, 4). Notwithstanding, the VisuMax 500 Hz femtosecond laser (Carl Zeiss Meditec AG, Jena, Germany), which is the laser platform for flapless SMILE, has certain limitations (5), such as suction loss. Another limitation of SMILE with the VisuMax 500 is that there is no software option to control centration and adjust for cyclotorsion which can cause decentration and cyclotorsion misalignment and lead to suboptimal refractive outcomes.

The second generation of KLEx surgery named SMILE Pro was applied in clinical practice in the year 2023 (6). The VisuMax 800 femtosecond laser (Carl Zeiss Meditec AG, Jena, Germany), the laser platform of SMILE Pro, is claimed to offer new advances by incorporating automated centration and cyclotorsion control tools into the software in surgical performance (7). In addition, the upgraded femtosecond laser pulse frequency of 2 MHz in VisuMax 800 offers a reduced refractive lenticule creation time of less than 10 s (8), which is significantly shorter than that of VisuMax 500 with 500 kHz. This shorter laser delineation time is expected to further reduce the possibility of suction loss. Early reports of SMILE Pro with the VisuMax 800 have shown excellent visual and refractive outcomes (9–15). However, no comprehensive meta-analysis has compared the clinical outcomes using the VisuMax 800 and VisuMax 500.

In this review, we aim to compile all relevant data to evaluate the postoperative visual and refractive outcomes between the first and second-generation KLEx surgeries. This research is considered to be useful in helping ophthalmologists choose the best refractive surgery technique for myopia and astigmatism eyes.

## 2 Methods

### 2.1 Protocol and registration

According to the Preferred Reporting Items for Systematic Reviews and Meta-analyses 2020 guidelines for systematic reviews and meta-analyses (16). This study was registered with PROSPERO (CRD420261294465). Meta-analyses were performed according to the PRISMA guidelines (17). This meta-analysis is based on publicly available, fully anonymized data and does not involve human participants or their personal data, hence Ethics, Consent to Participate, and Consent to Publish declarations are not applicable.

### 2.2 Search strategy

Two researchers independently and comprehensively searched the following electronic databases for records that compared SMILE using VisuMax 800 (SMILE Pro group) and VisuMax 500 (SMILE group) for correcting myopia and myopic astigmatism: PubMed, EMBASE and the Cochrane Library. No date restrictions in the electronic literature search were used, and the last search was run on Feb 22, 2026. Manual search brought us back an additional article that was included afterwards. The specific retrieval strategy was shown in Supplementary file 1.

### 2.3 Inclusion and exclusion criteria

The inclusion criteria were: (1) controlled clinical studies; (2) original papers reporting independent data; (3) all eyes treated with SMILE either using VisuMax 800 or VisuMax 500; (4) all adult patients with stable myopia or myopic astigmatism and without systemic or localized eye disease; (5) studies with at least one primary or secondary outcome reported; and (6) a follow-up period of at least 1 month. The exclusion criteria were: (1) noncomparative studies, (2) nonhuman investigations, (3) nonclinical studies, (4) reports with incorrect or incomplete data and (5) articles without outcomes of interest.

### 2.4 Quality assessment

Two reviewers evaluated the quality of the included literature independently. The risk of bias for the randomized controlled trials (RCTs) was evaluated using the Cochrane Risk-of-Bias Tool (18). whereas the quality of the nonrandomized controlled trials was assessed using the Newcastle-Ottawa Scale (NOS) (19). Grading of Recommendations Assessment, Development and Evaluation (GRADE) (20) working group framework was used to evaluate the certainty of findings.

### 2.5 Outcome measures

Data from the original article were extracted independently by two reviewers. The following outcome parameters were extracted.

### 2.6 Primary outcomes

The primary outcome measures were postoperative safety, efficacy, predictability and accuracy, while the safety measure were the postoperative mean logMAR corrected distance visual acuity (CDVA) and the eyes loss of one or more lines of CDVA. The percentage of eyes with an uncorrected distance visual acuity (UDVA) of 20/20 or better and the mean logMAR UDVA were the efficacy measures. The predictability and accuracy measures included the percentage of eyes within ±0.5 diopters (D) of the target refraction, the mean postoperative spherical equivalent (SE) and the postoperative refractive cylinder.

### 2.7 Secondary outcome

The secondary outcomes included the postoperative high-order aberrations (HOAs) parameters, including spherical aberration (SA), coma, total higher-order aberrations (HOAs). The vector analysis parameters included target induced astigmatism (TIA) vector, surgically induced astigmatism (SIA) vector, difference vector (DV), angle of error (AE), |AE|, correction index (CI, SIA/TIA), the magnitude of error (ME, the arithmetic difference between the magnitudes of the SIA and TIA), and index of success (IOS, DV/TIA).

### 2.8 Data extraction

The data were extracted independently by two reviewers, and any disagreement was resolved by discussion until a consensus was reached. Unclear information was clarified as recommended by the Cochrane Handbook (21). The following data were extracted: author, year of publication, study period, number of participants included in the study, original inclusion criteria, written language, preoperative spherical equivalence, femtosecond laser platforms, follow-up time and outcome data. Two reviewers extracted the data independently from the retrieved studies, screened the titles and the abstracts, assessed whether the potentially relevant reports were a complete manuscript and summarized the results. If there were multiple reports for a particular study, only data from the most recent or representative publications were extracted.

### 2.9 Statistical analysis

In the comparative analysis of the research results, for dichotomous measures, odds ratios (OR) and corresponding 95% confidence intervals (CI) were used. Additionally, the mean difference (MD) and corresponding 95% CI were used to evaluate the continuous data. If the 95% CI crosses zero (for MD) or 1.0 (for OR), no significant difference was considered. Heterogeneity $I^{2}\geq 50\%$ went through “*leave*-one-out” sensitivity analysis. If no single driver, then went through subgroup analysis. The statistical analyses were performed using the open-source R program (Version 4.5.1). *p*-values <0.05 were considered to be statistically significant.

Publication bias was assessed by visual inspection of funnel plots; Egger's and Begg's tests were planned only for outcomes with at least 10 studies. Statistical significance was defined as a P<0.05.

## 3 Results

### 3.1 Result of the search

The search was conducted on Feb 22, 2026 and identified a total of 837 records. Duplicate records removed (n=204). Studies not pertinent to SMILE: 613. No comparison of outcomes:12. A manual search included an additional article, and ultimately, 9 non-randomized comparative studies (NRCSs) articles were included (22–30). Figure 1 showed a flow diagram of the included and excluded studies.

**Figure 1:**
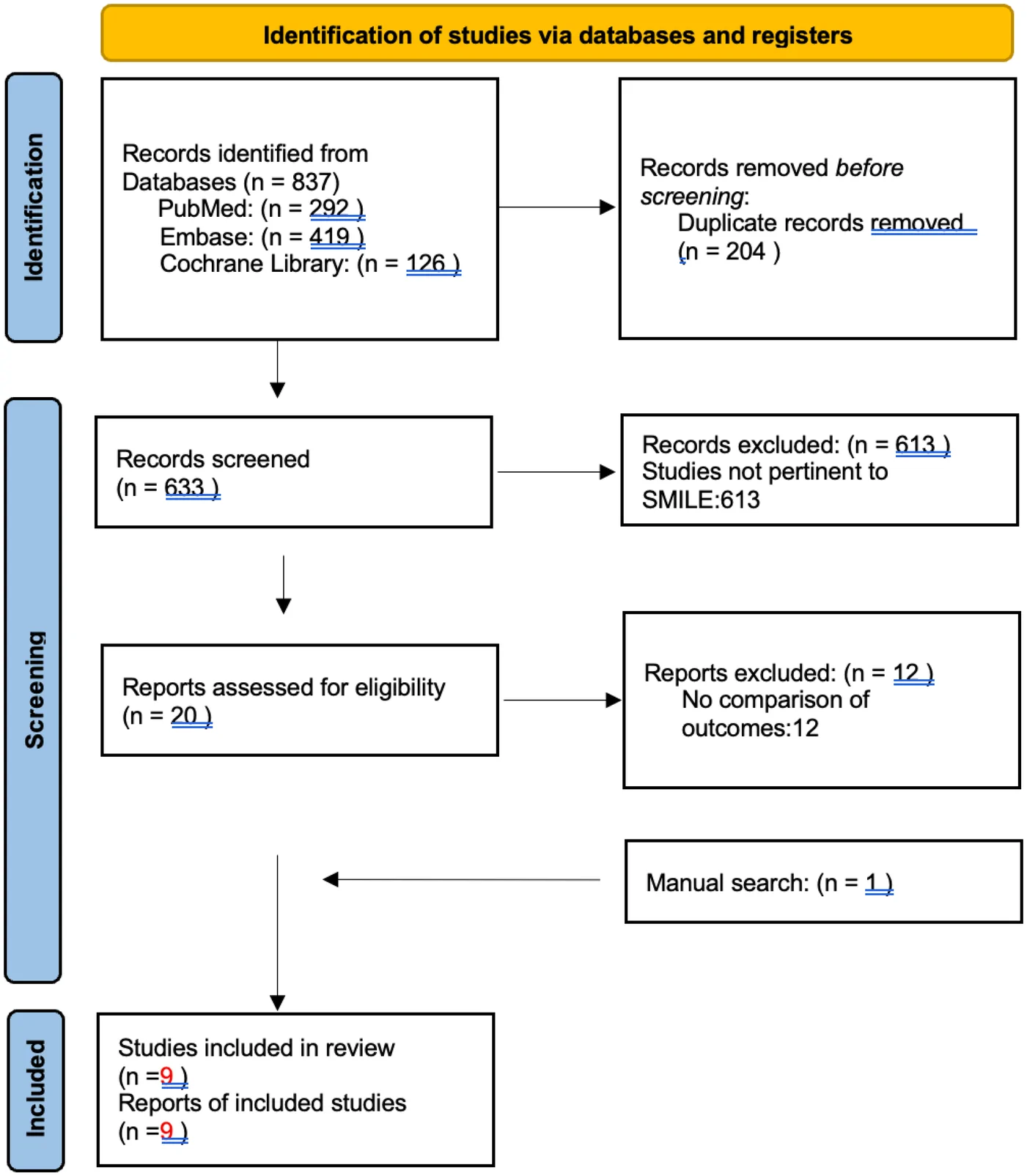
Flow chart depicting the selection of included studies.

### 3.2 Study characteristics and quality

Table 1 summarized the characteristics and quality of the 9 studies that met all inclusion criteria (22–30). All of the 9 selected studies were NRCSs with a total of 1,692 eyes. Although multiple publications from the same research group were included, they were used to extract different outcome measures rather than duplicate data for the same analysis. Potentially overlapping study populations cannot be entirely ruled out. All studies were published between 2024 and 2025. The follow-up period ranged from 1 to 6 months. All nine NRCSs were of relatively low risk of bias, scoring higher than 6 points on the NOS (Table 2).

**Table 1:** Characteristics of the included studies.

ID	References	Year	Design	Follow-up (months)	Language	Laser Platform	No. of eyes	Mean age (y)	Gender (%, female)	Preoperative SE(D)
1	Yoo et al. (22)	2024	RP	6	English	VISUMAX 800	50	24.5±5.3	60	NA
						VISUMAX 500	100	25.1±5.4	56	NA
2	Varman et al. (23)	2024	P	3	English	VISUMAX 800	105	28.48±6.24	48	−4.80±2.24
						VISUMAX 500	115	29.75±5.82	45	−4.82±2.16
3	Davis et al. (24)	2025	RP	3	English	VISUMAX 800	42	33.8±6.3	47.6	−5.42±1.58
						VISUMAX 500	45	32.4±7.8	47.8	−5.43±1.78
4	Lee et al. (25)	2024	RP	3	English	VISUMAX 800	35	33.37±8.47	65.7	−6.57±2.49
						VISUMAX 500	31	32.38±7.73	58	−5.79±2.72
5	Kim et al. (26)	2025	RP	3	English	VISUMAX 800	100	26.24±5.56	40	−4.57±1.6
						VISUMAX 500	100	26.14±4.27	46	−4.65±1.86
6	Ganesh et al. (27)	2025	P	3	English	VISUMAX 800	30	26.3±4.49	60	−3.85±1.86
						VISUMAX 500	30	26.3±4.49	63.3	−3.82±1.95
7	Chung et al. (28)	2025	RP	1	English	VISUMAX 800	45	32	88	−5.56
						VISUMAX 500	42	36	87.5	−6.02
8	Lee et al. (29)	2024	RP	3	English	VISUMAX 800	80	33.38±8.87	60	−5.49±2.31
						VISUMAX 500	80	31.52±7.42	52.5	−5.24±2.26
9	Kim et al. (30)	2025	RP	2	English	VISUMAX 800	169	24.0±5.6	50.6	−4.00±1.53
						VISUMAX 500	493	23.2±4.6	63.5	−4.09±1.61

SE = spherical equivalent, P = prospective comparative study, RP = retrospective comparative study, Y = year, NA = data not available.

**Table 2:** Risk of bias assessment based on the Newcastle-Ottawa scale for nonrandomized studies.

Author, year	Selection	Comparability	Outcome	Score
Yoo et al. (22)	4	2	3	9
Varman et al. (23)	4	2	3	9
Davis et al. (24)	4	2	3	9
Lee(a) et al. (25)	4	2	3	9
Kim(a) et al. (26)	4	2	3	9
Ganesh et al. (27)	4	2	3	9
Chung et al. (28)	4	1	2	7
Lee(b) et al. (29)	4	2	3	9
Kim(b) et al. (30)	4	2	2	8

### 3.3 Primary outcomes

Efficacy. Seven studies (22–26, 29, 30) evaluated postoperative logMAR UDVA. The results showed high heterogeneity among the studies ($I^{2}=99\%$). Using a random effects model, the difference in UDVA between the two groups was not statistically significant (MD = 0.02; 95% CI, −0.03 to 0.07) (Figure 2). Of the 9 studies, eight reported the proportions of eyes achieving a UDVA of 20/20 or better (22–28, 30). A total of 95.6% and 94.1% of eyes achieved a UDVA of 20/20 or better in the SMILE and SMILE Pro groups, respectively. Analysis of these data showed no difference between the two groups (OR, 0.90; 95% CI, 0.43 to 1.85) (Figure 3). All eyes were ≥20/40 in each group. Two studies (24, 26) evaluated the efficacy index. Overall, efficacy index was nearly identical between two groups with no heterogeneity (MD = 0.02; 95% CI, −0.00 to 0.04) (Figure 4A).

**Figure 2:**
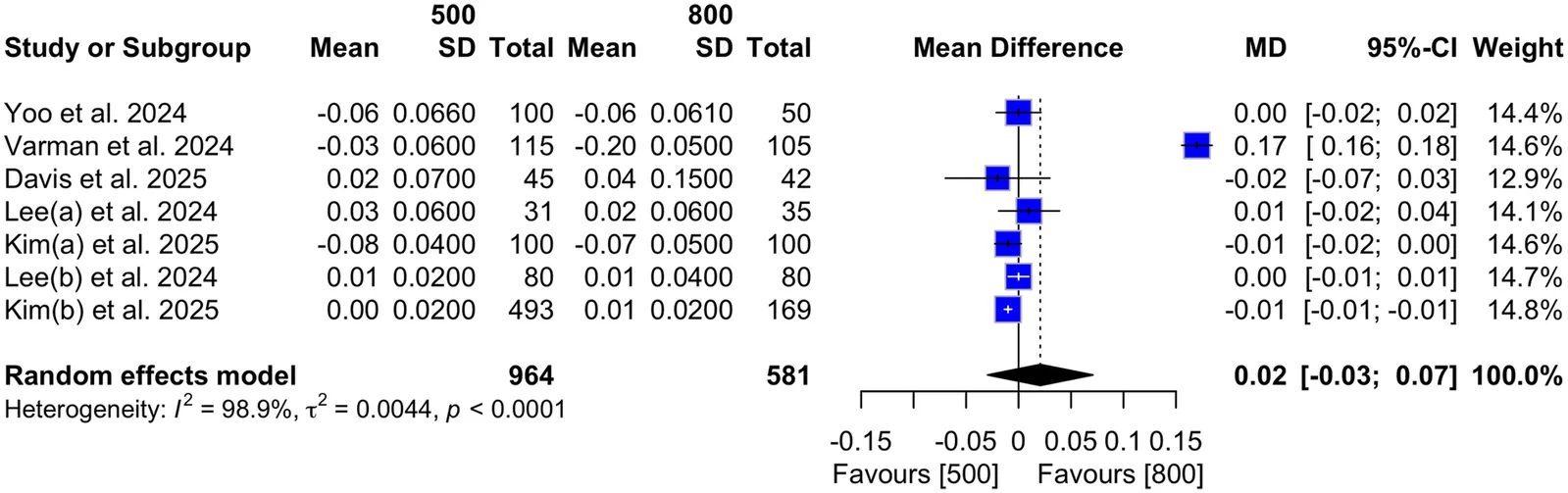
Forest plot of comparison on postoperative logMAR UDVA.

**Figure 3:**
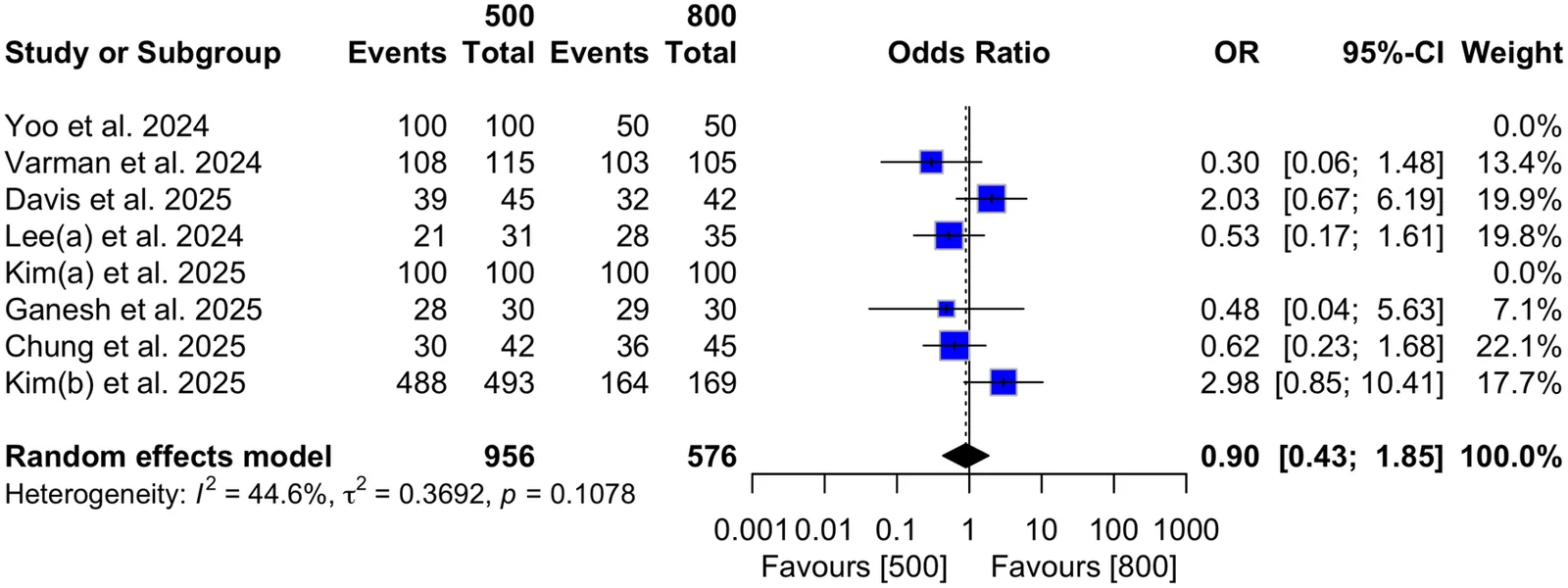
Forest plot of comparison on UDVA of 20/20 or better.

**Figure 4:**
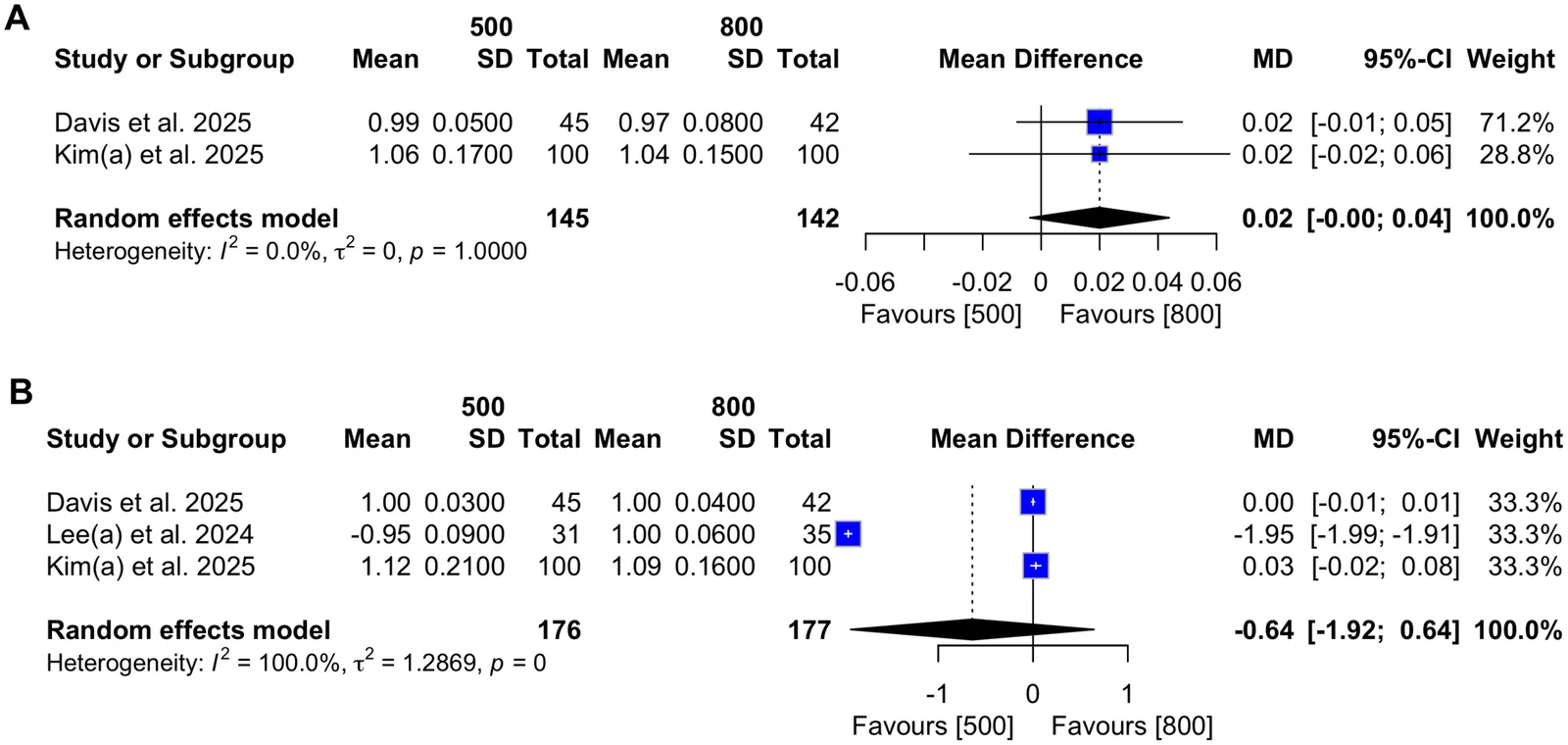
Forest plot of comparison on efficacy index and safety index.

Safety. The percentage of eyes gained one or more lines of CDVA was reported in 6 studies (22–27). Only 34.9% of the eyes gained one or more lines of CDVA in the SMILE group, and 19.9% of the eyes in the SMILE Pro group. The forest plots showed no significant difference between the two groups (OR, 1.77; 95% CI, 0.85 to 3.66) (Figure 5A).The percentage of eyes gained two or more lines of CDVA was reported in 6 studies (22–27). Only 4.28% of the eyes gained two or more lines of CDVA in the SMILE group, and 2.49% of the eyes in the SMILE Pro group. The forest plots showed no significant difference between the two groups (OR, 0.95; 95% CI, 0.15 to 5.96) (Figure 5B). The percentage of eyes no change of CDVA was reported in 6 studies (22–27). Only 55.1% of the eyes was reported no change of CDVA in the SMILE group, and 74.0% of the eyes in the SMILE Pro group. The forest plots showed significant difference between the two groups (OR, 0.47; 95% CI, 0.25 to 0.86) (Figure 5C). The percentage of eyes losing one line of CDVA was reported in 7 studies (22–28). Only 4.75% of the eyes lost one line of CDVA in the SMILE group, and 3.19% of the eyes in the SMILE Pro group. The forest plots showed no significant difference between the two groups (OR, 1.53; 95% CI, 0.73 to 3.21) (Figure 5D). Only single event of losing two or more lines of CDVA was recorded in SMILE group. Three studies (24–26) evaluated the safety index. Overall, there was no statistically significant difference in safety index between the two groups, although heterogeneity was considerable (I² = 100%) (MD = −0.64; 95% CI, −1.92 to 0.64) (Figure 4B). As for adverse events, five (23, 26–29) reported postoperative complications. There was a single case (0.27%) (28) where loss of suction occurred in SMILE group (367 eyes) and none in SMILE Pro group (360 eyes).

**Figure 5:**
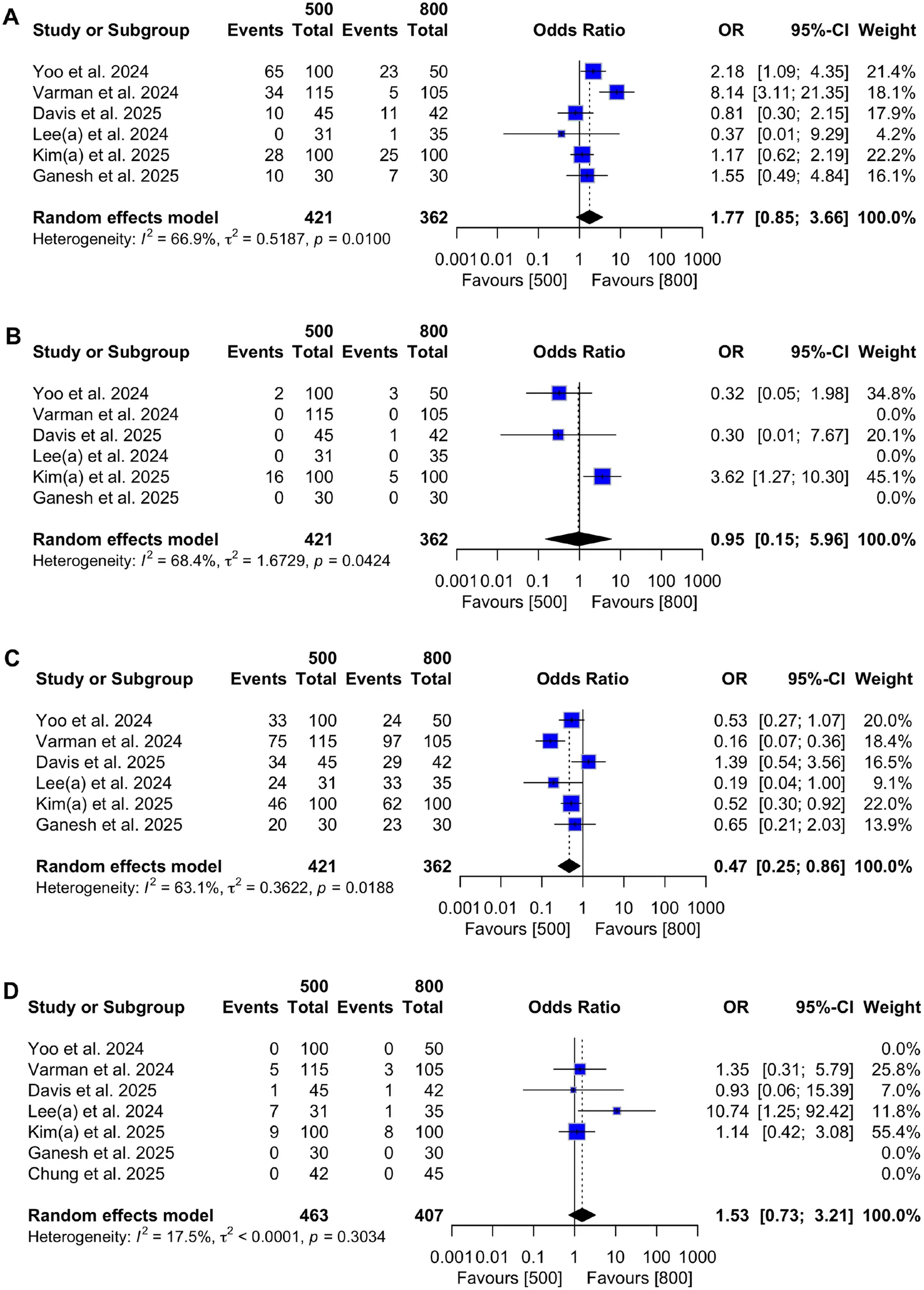
Forest plot of comparison on gaining or loss of CDVA [**(A)**: gaining one or more lines, **(B)**: gaining two or more lines, **(C)**: no change, **(D)**: losing one or more lines].

Predictability. Six studies (23–26, 29, 30) reported the postoperative refractive SE (MD = −0.01; 95% CI, −0.12 to 0.10) (Figure 6A) with no significant difference. Data were collected from eight studies (22–28, 30) to report postoperative refraction SE within ±0.5 D of the target refraction, and the forest plot for this outcome showed no significant difference between the two groups (OR, 0.58; 95% CI, 0.29 to 1.14) (Figure 7A). Six studies (22–26, 30) reported the postoperative refractive cylinder (MD = −0.06; 95% CI, −0.13 to 0.00) (Figure 6B) and SMILE group showed a marginally greater residual cylinder than the SMILE Pro group. Data were collected from six studies (22, 23, 25–27, 30) to report postoperative refraction Cylinder within ≤0.5 D of the target refraction, and the forest plot for this outcome showed no significant difference between the two groups (OR, 0.66; 95% CI, 0.38 to 1.14) (Figure 7B).

**Figure 6:**
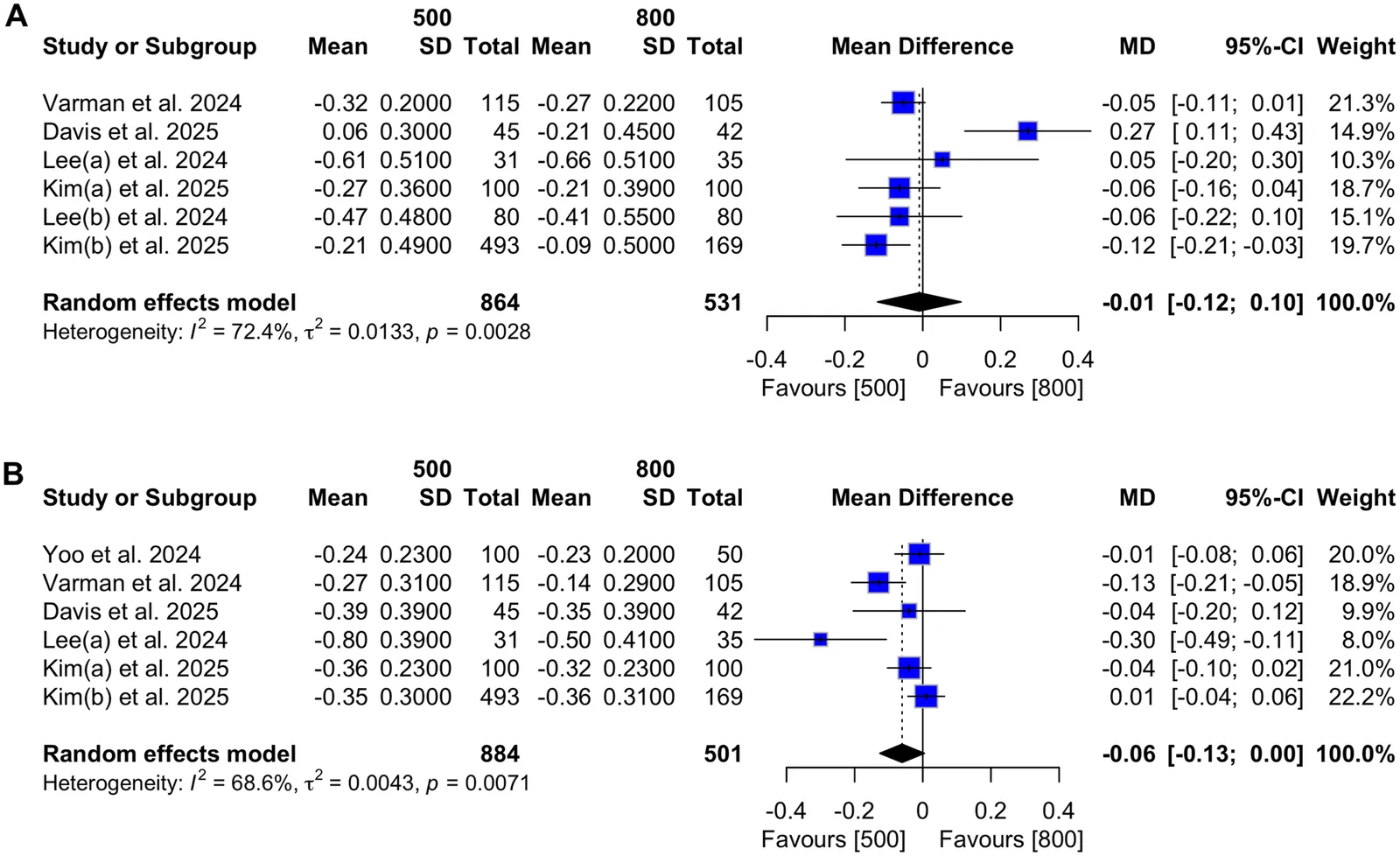
Forest plot of comparison on postoperative refractive SE **(A)** and cylinder **(B)**.

**Figure 7:**
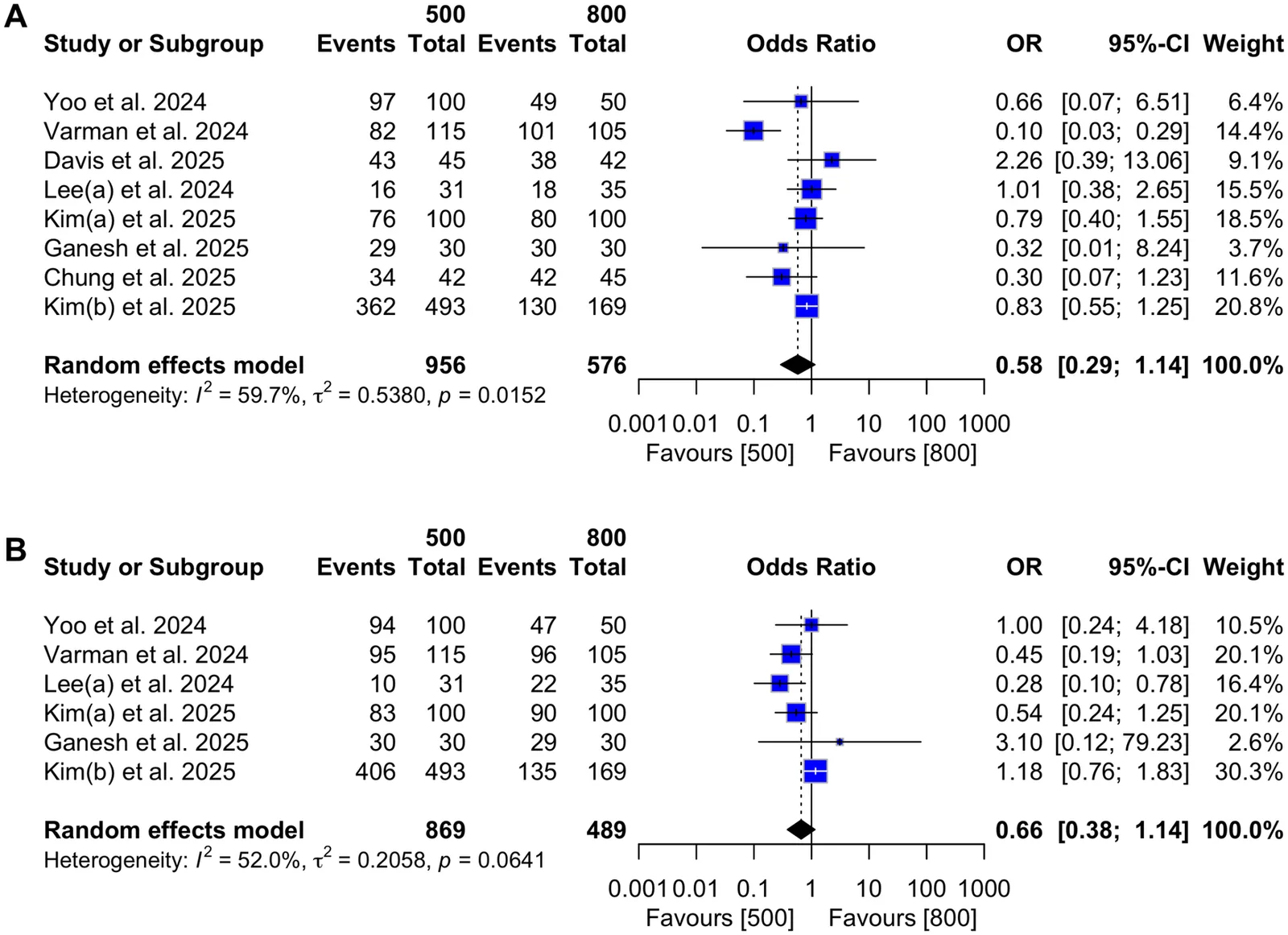
Forest plot of comparison on postoperative refraction SE within ±0.5D of the target refraction **(A)** and Cylinder within ≤0.5D **(B)**.

### 3.4 Secondary outcomes

Vector analysis. Of nine studies included, five (23–26, 30) evaluated outcomes of SIA. There was significant heterogeneity among the studies (I^2^ = 86.7%), so the random effects model was used for analysis. The results showed that there was no statistically significant difference in SIA between the two groups (MD = −0.08; 95% CI, −0.36 to 0.20) (Figure 8A). Five studies (23–26, 30) evaluated outcomes of TIA. The results showed that there was no statistically significant difference in TIA between the two groups (MD = -0.03; 95% CI, −0.33 to 0.28) (Figure 8B). Four studies (23, 24, 26, 30) evaluated outcomes of AE. The results showed that there was no statistically significant difference in AE between the two groups (MD = 0.59; 95% CI, −0.23 to 1.42) (Figure 8C). Five studies (23–26, 30) evaluated outcomes of |AE|. The results showed that there was statistically significant difference in |AE| between the two groups (MD = 4.29; 95% CI, 2.34 to 6.24) (Figure 8D). For other parameters including DV, CI, ME, IOS, no pooled MD or pooled OR was calculated due to limited data.

**Figure 8:**
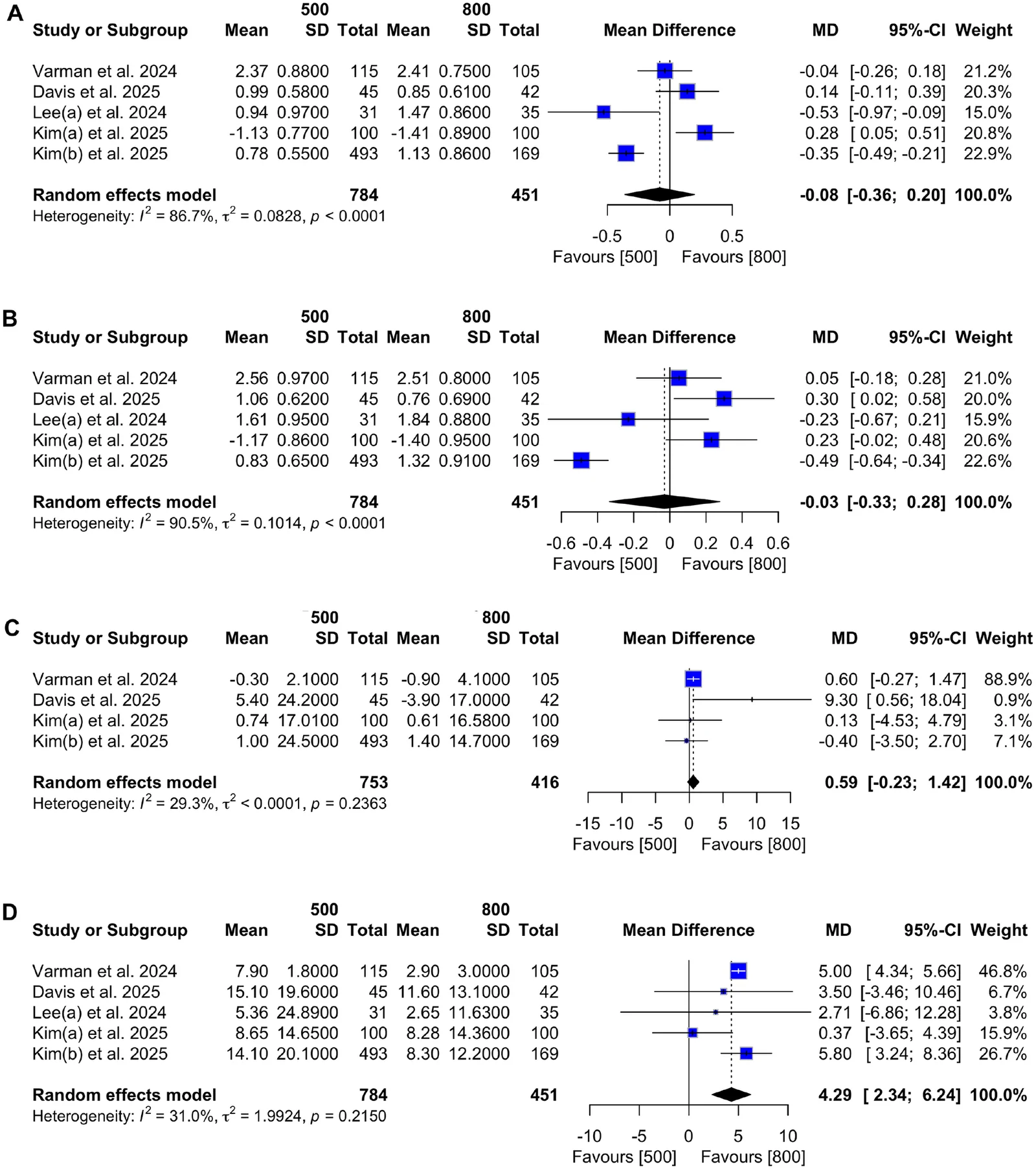
Forest plot of comparison on vector analysis parameters [**(A)**: SIA, **(B)**: TIA, **(C)**: AE, **(D)**: |AE|].

Aberrations. Three studies (22, 26, 27) evaluated spherical aberration(SA) outcomes. The results showed that the effect of SMILE Pro on SA correction was not statistically significant compared with the SMILE group (MD = −0.08; 95% CI, −0.21 to 0.06) (Figure 9A). Two studies (22, 26) evaluated vertical coma and horizontal coma outcomes. The results showed that the effect of SMILE Pro on vertical and horizontal coma correction was not statistically significant compared with the SMILE group (MD = −0.03; 95% CI, −0.19 to 0.12; MD = 0.01; 95% CI, −0.03 to 0.04; respectively) (Figures 9B,C). Three studies (22, 26, 27) evaluated HOAs outcomes. The results showed that the effect of SMILE Pro on HOAs correction was not statistically significant compared with the SMILE group (MD = 0.03; 95% CI, −0.01 to 0.07) (Figure 9D), suggesting that using SMILE Pro had no significant effect on higher-order aberrations.

**Figure 9:**
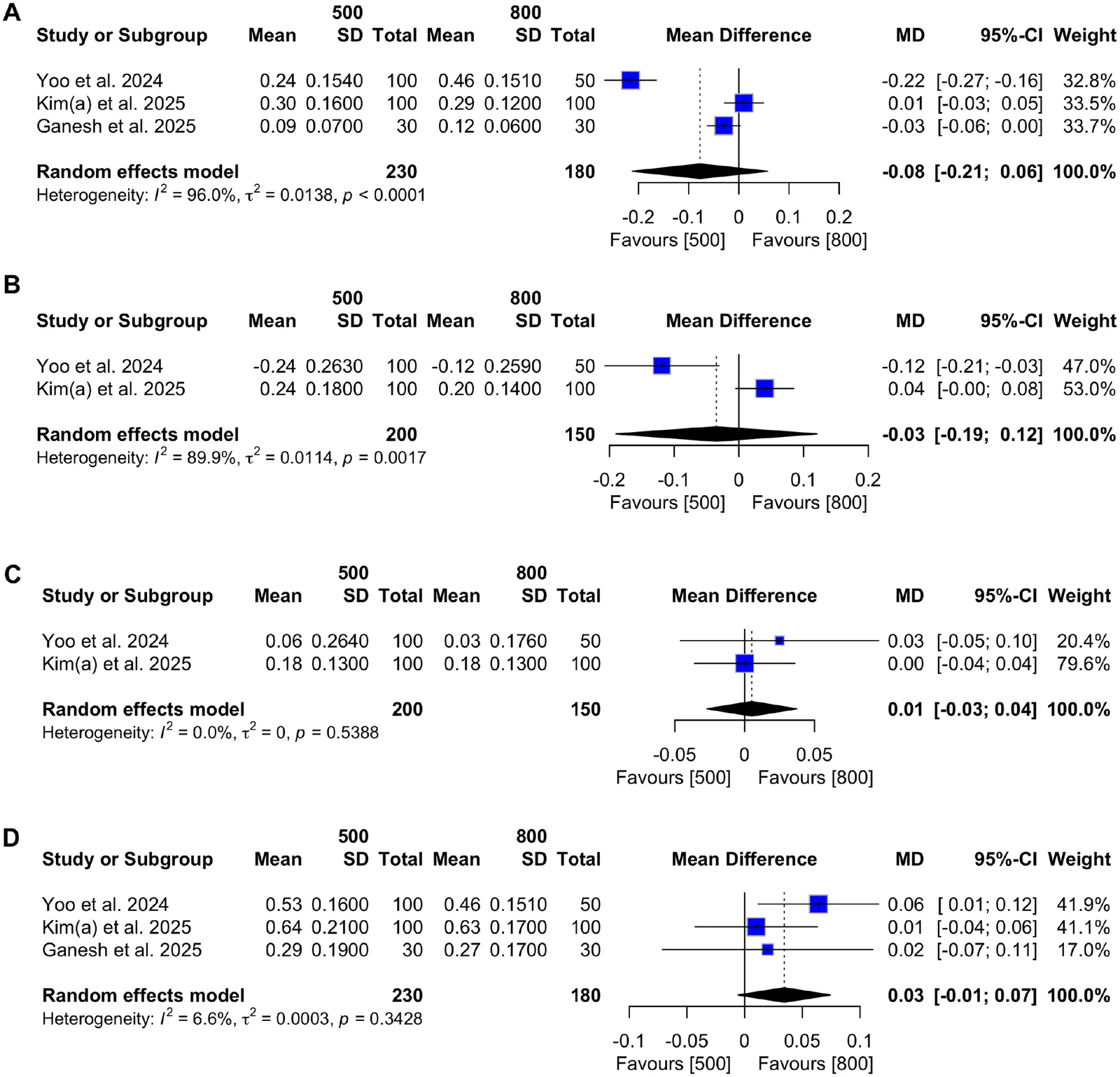
Forest plot of comparison on aberrations parameters [**(A)**: spherical aberration, **(B)**: vertical coma, **(C)**: horizontal coma, **(D)**: HOAs].

### 3.5 Publication bias test

Funnel plots were generated for all outcomes with three or more studies (supplementary file 3). Formal statistical tests (Egger’s and Begg’s) require a minimum of 10 studies and could not be performed for any outcome. With only 9 included studies, the risk of publication bias cannot be reliably assessed.

## 4 Discussion

Given the popularity of SMILE and the latest upgrade of the SMILE platform, we performed a meta-analysis to compare the outcomes of SMILE and SMILE Pro based on the published comparative articles for myopia and myopic astigmatism. The pooled results showed that no significant difference was discovered in the proportion of eyes with safety, efficacy and optical quality. A significant difference existed in astigmatism correction.

As far as we know, it was the first study to compare SMILE and SMILE Pro using Meta-analysis. While current evidence may not yet establish definitive clinical protocols, our findings could offer practical references for optimizing therapeutic decision-making in clinical practice. Particularly amidst myopia increasing incidence over the past few decades, myopia remains an important public health issue in various areas of the world (31, 32). However, there was a high degree of statistical heterogeneity in some of the pooled analysis. We attempted to use leave-one-out sensitivity analysis and subgroup analysis to exclude the possibility of heterogeneity, but no satisfactory results were obtained, and some unexplained heterogeneity remained. So, it should be emphasized that the conclusions drawn herein require cautious interpretation given the inherent limitations of available studies and the potential biases in the review process.

In terms of efficacy, both SMILE group and SMILE Pro group brought good visual acuity in patients with myopia and myopic astigmatism. All of eyes achieved 20/40 or better in both groups. The forest plot indicated no significant differences in postoperative logMAR UDVA or the proportion of eyes having UDVA of 20/20 or better between the SMILE and SMILE Pro groups. However, there was significant heterogeneity in postoperative logMAR UDVA. Leave-one-out sensitivity analysis demonstrated that the study of Varman et al. (23) was the cause of statistical heterogeneity in the postoperative logMAR UDVA, and when the study of Varman et al. (23) was excluded, I^2^ dropped to 17.1% and the pooled estimate was −0.0066. The difference was statistically significant, however, it’s not clinically meaningful. In addition, all eyes logMAR UDVA results were inconsistent with sub-cohort results in the study of Varman et al. (23), which likely had a data quality issue. Efficacy Index was nearly identical between two groups with no heterogeneity. However, the clinical significance cannot be determined solely from the 95% CI (−0.00, 0.04); it required comprehensive analysis incorporating clinical context.

In terms of safety, only the study of Kim et al. (26) recorded a single event of losing two or more lines of CDVA in SMILE group, making meaningful pooling impossible and the estimated OR not reliable. The forest plot indicated no significant differences in losing one line, gain of 1 line or gain of 2 lines between the SMILE and SMILE Pro groups. However, the forest plot revealed that the percentage of no change of CDVA was significant difference. SMILE group were significantly less likely to maintain unchanged CDVA compared to SMILE Pro group. Leave-one-out sensitivity analysis for the percentage of no change in CDVA showed that when the study of Varman et al. (23) was removed, I^2^ dropped to 23.3% and the pooled OR to 0.5918, but the pooling results remained unchanged and the difference was still statistically significant. In addition, as for safety index, there was no significant difference. Although too few studies for leave-one-out analysis, the study of Lee et al. (25) may be the outlier distorting the pooled estimate for safety index. For CDVA, no pooled MD was calculated due to limited data.

Large studies had demonstrated that the incidence of suction loss during SMILE using Visumax 500 ranges from 0.17 to 5.06% of patients (33–35). Consistent with this finding, our study indicated that suction loss rate was 0.27% in SMILE group and none in SMILE Pro group. The greater reduction in suction loss rate with the VisuMax 800 may be attributable to the significantly faster laser time of less than 10 s (8). Given there is limited long-term data on SMILE Pro, further multicentre studies with larger sample sizes and longer follow-up would be required to verify this.

In terms of predictability and accuracy, both groups achieved excellent postoperative residual error in the 9 included studies. We found no significant differences between the SMILE and SMILE Pro groups with regard to the postoperative refractive SE and the proportion of postoperative refraction within ±0.50D. However, both of them were significant heterogeneity. Leave-one-out sensitivity analysis for the postoperative refractive SE showed removal of the study of Davis et al. (24) reduced I^2^ to 0%, but the result became statistically significant difference. The study of Davis et al. (24) showed the mean SE in SMILE Pro group was significantly more myopic than the SMILE group, which may be the outlier pulling the estimate toward SMILE Pro group. Without it, the remaining studies consistently favored SMILE group with no heterogeneity. Leave-one-out sensitivity analysis for the proportion of postoperative refraction within ±0.50 showed when the study of Varman et al. (23) was removed I^2^ drops to 0% and the pooled OR to 0.81, but the difference was still not statistically significant.

In terms of cylinder and vector analysis, the forest plot indicated no significant differences in Cyl≤0.50D. Leave-one-out sensitivity analysis for Cyl≤0.50D showed removal the study of Kim et al. (30) reduced I^2^ to 0% and the pooled OR to 0.4899, and is statistically significant difference. However, the study of Kim et al. (30) carried the largest sample size (about 39%), so its removal may not be justified. Certain parameters including TIA, SIA, and AE remained comparable between both groups. Of nine studies included, five evaluated outcomes of |AE|. SMILE group showed a marginally greater residual cylinder than the SMILE Pro group, which does not necessarily imply clinical significance. Leave-one-out sensitivity analysis showed heterogeneity was substantial but all studies except the study of Kim et al. (30) favored the SMILE group. The direction of effect was consistent even though magnitude varied. Subgroup analyses were performed on the primary outcomes with regard to the preoperative astigmatism values (low cylinder and high cylinder cohorts). There was significant difference between two subgroups. For the low cylinder cohort, there was no significant difference within group. For the high cylinder cohort, there was significant difference. These findings indicated VISUMAX 800 provided more accurate refractive cylinder outcomes when correcting more than 2 D of cylinder and nomograms should be adjusted to correct astigmatism based on the preoperative astigmatism values (23, 24).

The induction of higher order aberrations (HOAs) was a concern in corneal refractive surgery, as it could affect visual quality, leading to symptoms such as glare, halos, and reduced contrast sensitivity (36). A noteworthy discovery was that the forest plot indicated no significant differences in total HOAs, spherical aberration, vertical coma and horizontal coma. Although too few studies for leave-one-out analysis, the pooled estimate should be interpreted with caution.

In terms of publication bias, visual inspection revealed asymmetry in several outcomes, but this was primarily attributable to identified outlier studies and clinical heterogeneity rather than systematic publication bias.

This review has several limitations. Firstly, the pooled analyses exhibited significant heterogeneity, likely attributable to clinical diversity across studies including variability in myopia and myopic astigmatism severity, operator experience and follow-up times. Secondly, most studies reported in this review focused on visual outcomes without assessing patient-reported outcomes such as dry eye and visual disturbances. Other important parameters, like corneal biomechanics and epithelial mapping, were not evaluated. Thirdly, the evidence hierarchy remained suboptimal due to scarce randomized comparisons between SMILE group and SMILE Pro group. Future randomized multicentre studies could integrate both objective measures and subjective measures to further distinguish the strengths and limitations of one platform over another (37).

## 5 Conclusion

In conclusion, within the limitations of this study, we did not detect evidence of significant difference between the two surgical procedures, SMILE and SMILE Pro, used for the treatment of myopia and astigmatism. Further multicentre studies with larger sample sizes and longer follow-up are warranted to confirm VisuMax 800 system superior practical benefits in surgical workflow, refractive outcomes and patient comfort.

## Data Availability

The original contributions presented in the study are included in the article/supplementary material, further inquiries can be directed to the corresponding author/s.
