# Supplementary material for: Comparison of outcomes after SMILE using VISUMAX 800 and VISUMAX 500 for correcting myopia and myopic astigmatism: a systematic review and meta-analysis
Source: Front Med (Lausanne). 2026 Oct 7;13:1899883. doi: 10.3389/fmed.2026.1899883 (PMC13683969; doi:10.3389/fmed.2026.1899883)
Supplement: Supplementary file 1 [file Datasheet1.pdf]

1.Leave-One-Out Sensitivity Analysis for UDVA

Leave-One-Out Sensitivity Analysis for UDVA

Full model: MD = 0.0207, 95% CI [-0.0295; 0.0709], I-squared = 98.9%

Excluded Study	MD	95% CI Lower	95% CI Upper	I2 (%)
Yoo et al. 2024	0.0240	-0.0348	0.0828	99.1
Varman et al. 2024	-0.0066	-0.0122	-0.0009	17.1
Davis et al. 2025	0.0267	-0.0300	0.0835	99.1
Lee(a) et al. 2024	0.0223	-0.0367	0.0813	99.1
Kim(a) et al. 2025	0.0258	-0.0324	0.0841	99.1
Lee(b) et al. 2024	0.0241	-0.0348	0.0831	99.1
Kim(b) et al. 2025	0.0259	-0.0324	0.0842	98.9

2.Leave-One-Out and subgroup Sensitivity Analysis for Cylinder(D)

Leave-One-Out Sensitivity Analysis for Cylinder (D)

Full model: MD = -0.0607, 95% CI [-0.1263; 0.0048], I-squared = 68.6%

Excluded Study	MD	95% CI Lower	95% CI Upper	I2 (%)
Yoo et al. 2024	-0.0780	-0.1626	0.0066	73.6
Varman et al. 2024	-0.0303	-0.0784	0.0178	59.1
Davis et al. 2025	-0.0674	-0.1467	0.0120	74.8
Lee(a) et al. 2024	-0.0380	-0.0894	0.0134	53.7
Kim(a) et al. 2025	-0.0729	-0.1633	0.0174	74.8
Kim(b) et al. 2025	-0.0813	-0.1565	-0.0061	64.3

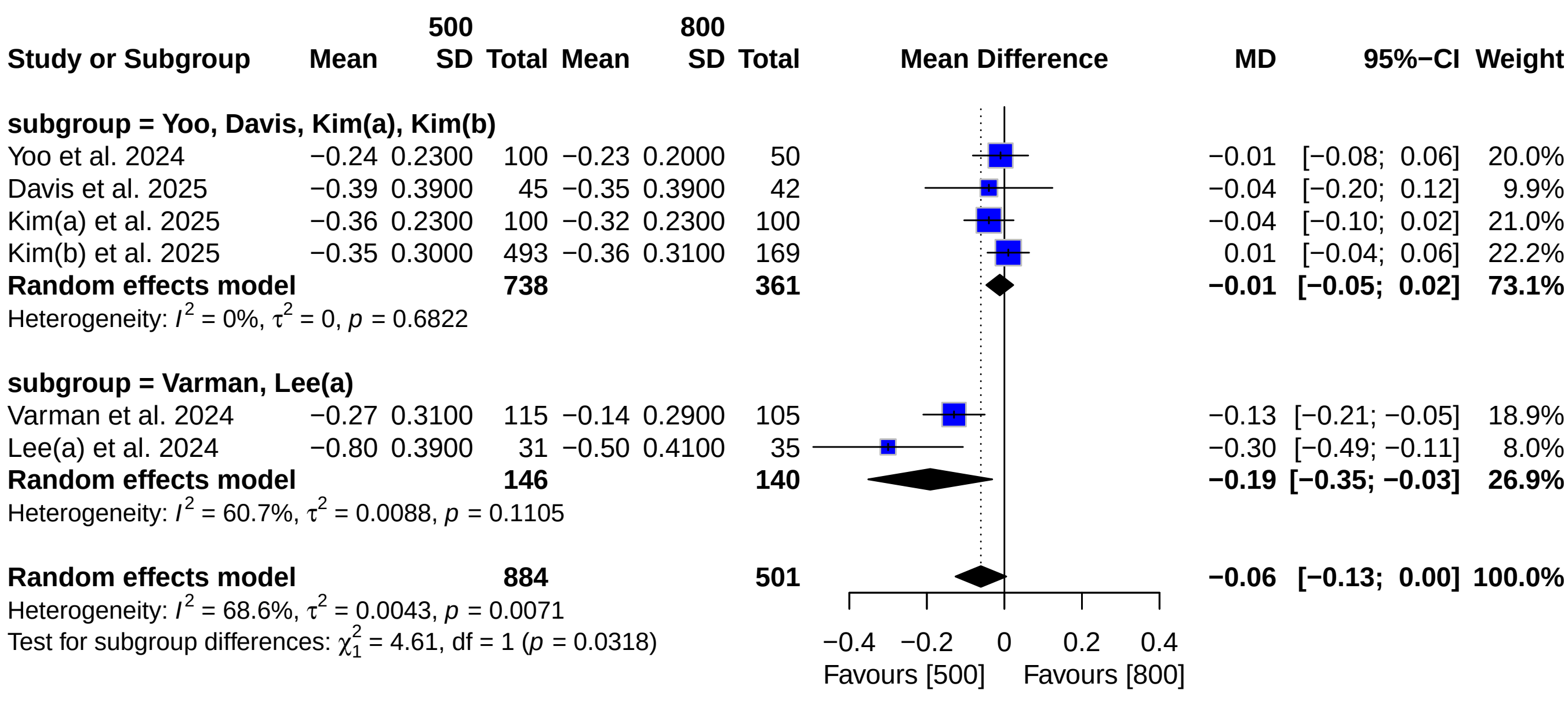

3.Leave-One-Out Sensitivity Analysis for SEQ(D)

Leave-One-Out Sensitivity Analysis for SEQ (D)

Full model: MD = -0.009, 95% CI [-0.1164; 0.0984], I-squared = 72.4%

Excluded Study	MD	95% CI Lower	95% CI Upper	I2 (%)
Varman et al. 2024	0.0053	-0.1329	0.1435	77.8
Davis et al. 2025	-0.0648	-0.1056	-0.0241	0.0
Lee(a) et al. 2024	-0.0143	-0.1367	0.1082	77.2
Kim(a) et al. 2025	0.0060	-0.1287	0.1406	77.8
Lee(b) et al. 2024	0.0034	-0.1273	0.1342	77.9
Kim(b) et al. 2025	0.0188	-0.1041	0.1416	72.4

4.Leave-One-Out Sensitivity Analysis for Cyl<=0.50D

Leave-One-Out Sensitivity Analysis for Cyl <= 0.50D

Full model: OR = 0.6614, 95% CI [0.3828; 1.1426], I-squared = 52%

Excluded Study	OR	95% CI Lower	95% CI Upper	I2 (%)
Yoo et al. 2024	0.6241	0.3371	1.1551	61.2
Varman et al. 2024	0.7263	0.3811	1.3841	52.1
Lee(a) et al. 2024	0.7891	0.4647	1.3399	34.4
Kim(a) et al. 2025	0.6883	0.3487	1.3587	58.1
Ganesh et al. 2025	0.6326	0.3607	1.1093	58.9
Kim(b) et al. 2025	0.4899	0.3043	0.7887	0.0

5.Leave-One-Out Sensitivity Analysis for SE within+/-0.50D

Leave-One-Out Sensitivity Analysis for SEQ within +/- 0.50D

Full model: OR = 0.5782, 95% CI [0.2922; 1.1441], I-squared = 59.7%

Excluded Study	OR	95% CI Lower	95% CI Upper	I2 (%)
Yoo et al. 2024	0.5708	0.2717	1.1988	65.4
Varman et al. 2024	0.8122	0.5953	1.1082	0.0
Davis et al. 2025	0.5060	0.2499	1.0246	61.4
Lee(a) et al. 2024	0.5200	0.2358	1.1467	64.1
Kim(a) et al. 2025	0.5367	0.2336	1.2326	65.0
Ganesh et al. 2025	0.5908	0.2895	1.2059	65.0
Chung et al. 2025	0.6280	0.2934	1.3441	62.4
Kim(b) et al. 2025	0.5264	0.2278	1.2165	61.9

6.Leave-One-Out Sensitivity Analysis for TIA

Leave-One-Out Sensitivity Analysis for TIA

Full model: MD = -0.0296, 95% CI [-0.3347; 0.2755], I-squared = 90.5%

Excluded Study	MD	95% CI Lower	95% CI Upper	I2 (%)
Varman et al. 2024	-0.0493	-0.4385	0.3399	92.2
Davis et al. 2025	-0.1121	-0.4458	0.2215	90.0
Lee(a) et al. 2024	0.0105	-0.3511	0.3720	92.8
Kim(a) et al. 2025	-0.0963	-0.4515	0.2589	90.4
Kim(b) et al. 2025	0.1331	-0.0365	0.3026	40.2

7.Leave-One-Out Sensitivity Analysis for SIA

Leave-One-Out Sensitivity Analysis for SIA

Full model: MD = -0.0812, 95% CI [-0.359; 0.1965], I-squared = 86.7%

Excluded Study	MD	95% CI Lower	95% CI Upper	I2 (%)
Varman et al. 2024	-0.0968	-0.4634	0.2698	89.8
Davis et al. 2025	-0.1397	-0.4753	0.1959	88.0
Lee(a) et al. 2024	-0.0043	-0.2806	0.2721	88.8
Kim(a) et al. 2025	-0.1726	-0.4454	0.1003	81.0
Kim(b) et al. 2025	0.0022	-0.2952	0.2996	73.8

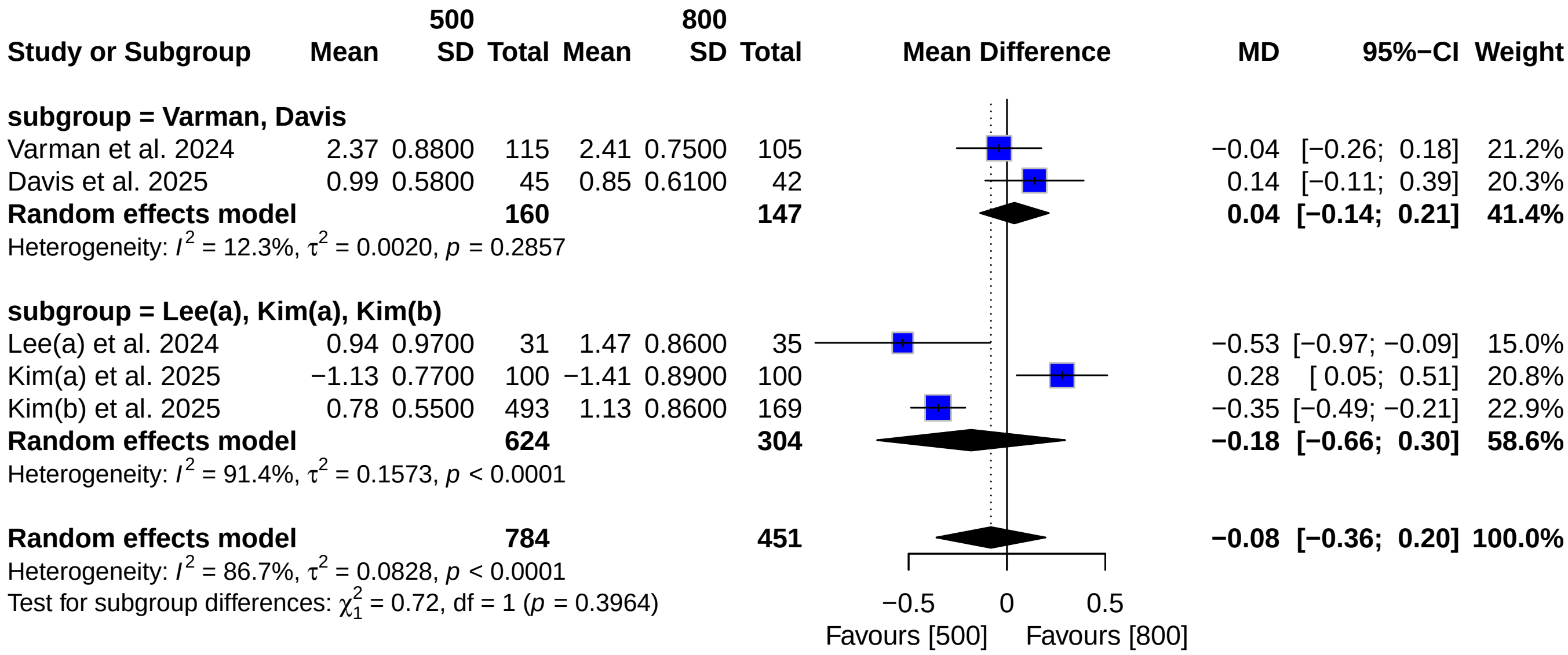

8.Leave-One-Out Sensitivity Analysis for No Change in CDVA

Leave-One-Out Sensitivity Analysis for No Change in CDVA

Full model: OR = 0.4662, 95% CI [0.2526; 0.8605], I-squared = 63.1%

Excluded Study	OR	95% CI Lower	95% CI Upper	I2 (%)
Yoo et al. 2024	0.4484	0.2052	0.9797	70.1
Varman et al. 2024	0.5918	0.4106	0.8530	23.3
Davis et al. 2025	0.3827	0.2204	0.6647	49.1
Lee(a) et al. 2024	0.5103	0.2648	0.9834	67.6
Kim(a) et al. 2025	0.4495	0.2031	0.9947	70.1
Ganesh et al. 2025	0.4393	0.2123	0.9093	69.8

9.Leave-One-Out Sensitivity Analysis for Gain of 1 Line of CDVA

Leave-One-Out Sensitivity Analysis for Gain of 1 Line of CDVA

Full model: OR = 1.7685, 95% CI [0.854; 3.6622], I-squared = 66.9%

Excluded Study	OR	95% CI Lower	95% CI Upper	I2 (%)
Yoo et al. 2024	1.6458	0.6401	4.2313	72.6
Varman et al. 2024	1.3556	0.8846	2.0775	0.0
Davis et al. 2025	2.1013	0.9487	4.6539	67.4

Excluded Study	OR	95% CI Lower	95% CI Upper	I2 (%)
Lee(a) et al. 2024	1.8972	0.8958	4.0178	71.8
Kim(a) et al. 2025	1.9590	0.7923	4.8438	68.3
Ganesh et al. 2025	1.7916	0.7287	4.4048	73.4
