# Supplementary material for: Comparison of outcomes after SMILE using VISUMAX 800 and VISUMAX 500 for correcting myopia and myopic astigmatism: a systematic review and meta-analysis
Source: Front Med (Lausanne). 2026 Oct 7;13:1899883. doi: 10.3389/fmed.2026.1899883 (PMC13683969; doi:10.3389/fmed.2026.1899883)
Supplement: Supplementary file 2 [file Datasheet2.pdf]

1. UDVA

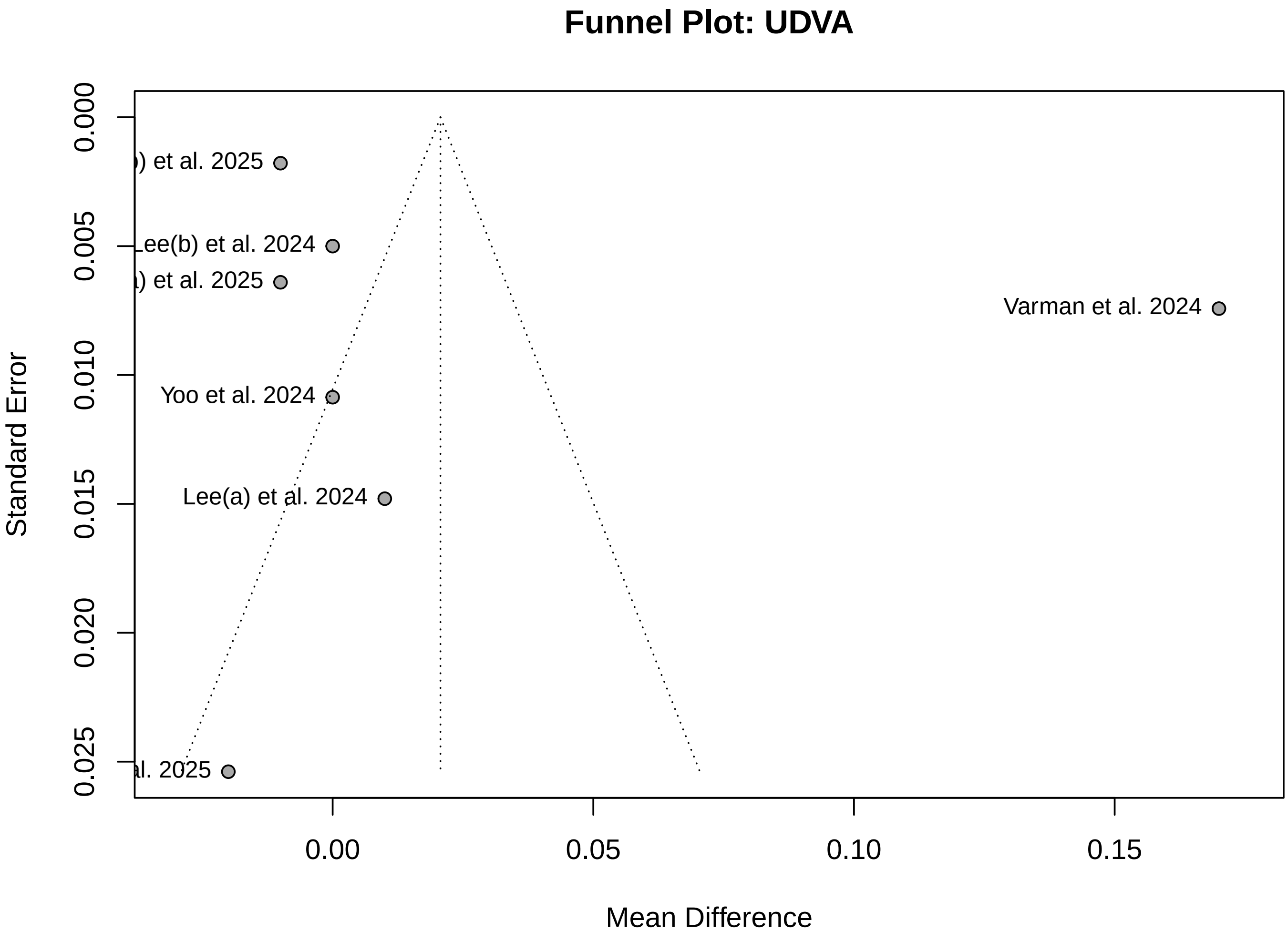

7 studies included. Egger's and Begg's tests require a minimum of 10 studies and were not performed.

2. UDVA 20/20 or Better

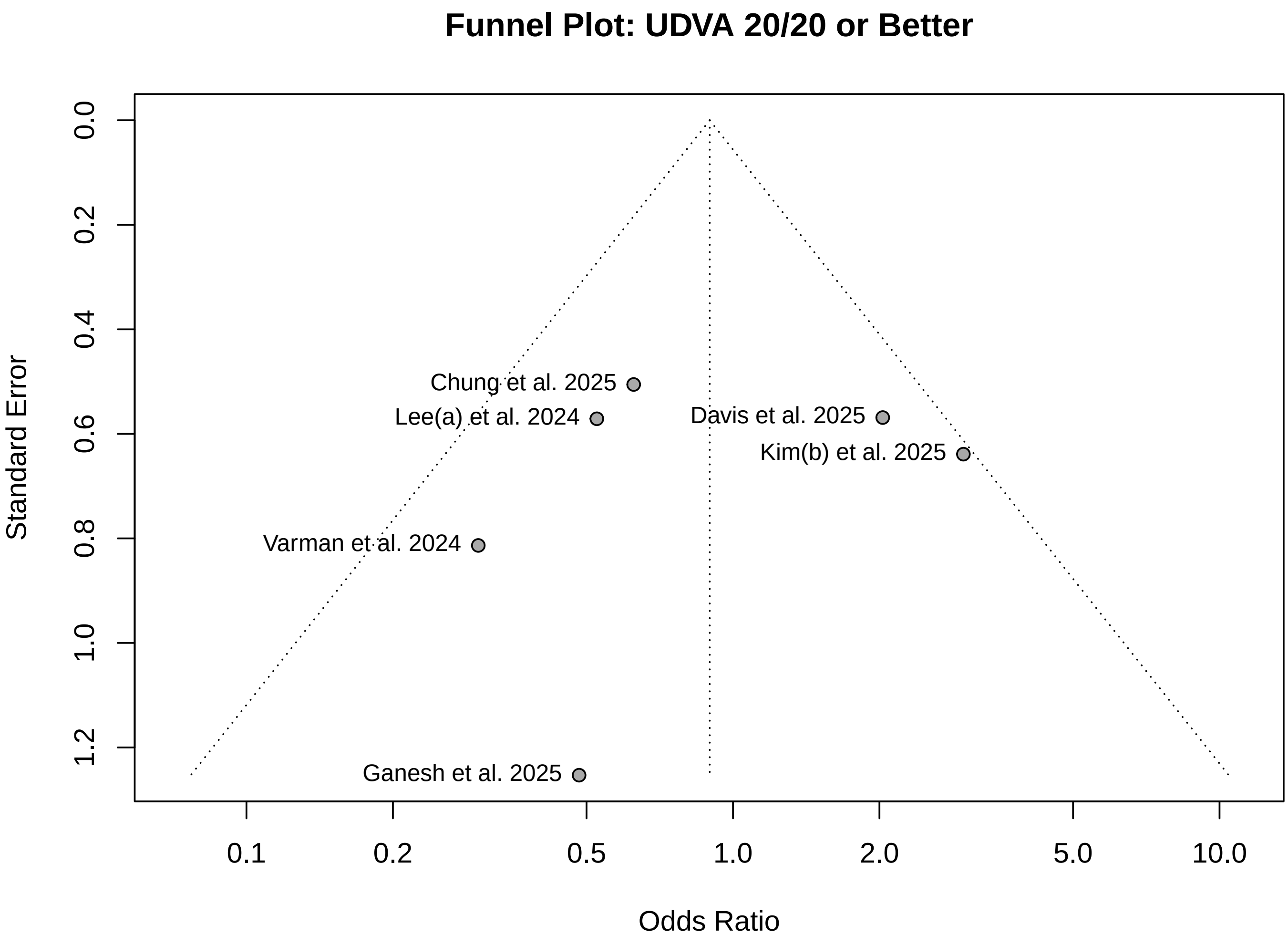

6 studies included. Egger's and Begg's tests require a minimum of 10 studies and were not performed.

3. Lost Two or More Lines of CDVA

Too few studies for publication bias assessment.

4. Sphere (D)

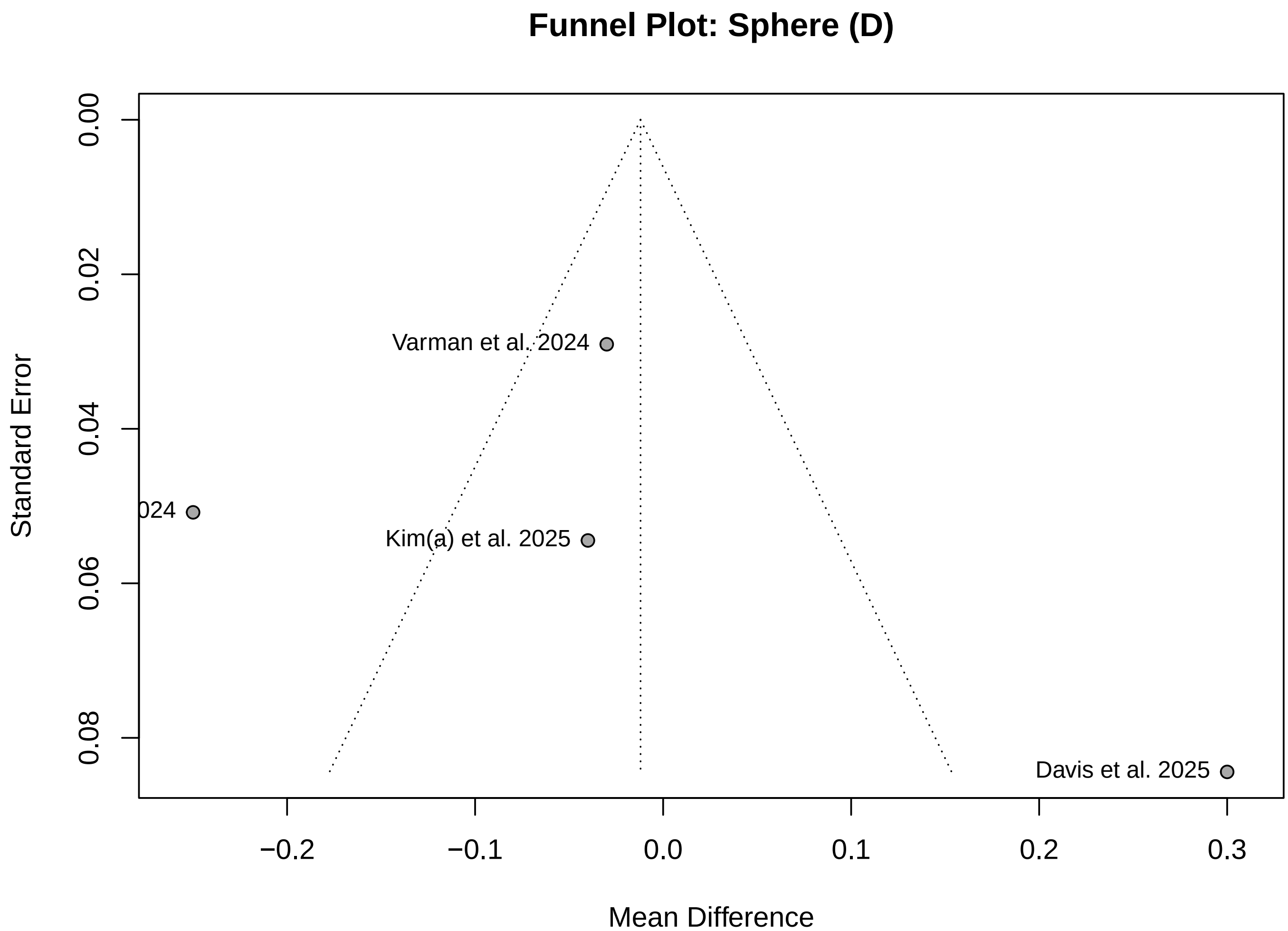

4 studies included. Egger's and Begg's tests require a minimum of 10 studies and were not performed.

5. Cylinder (D)

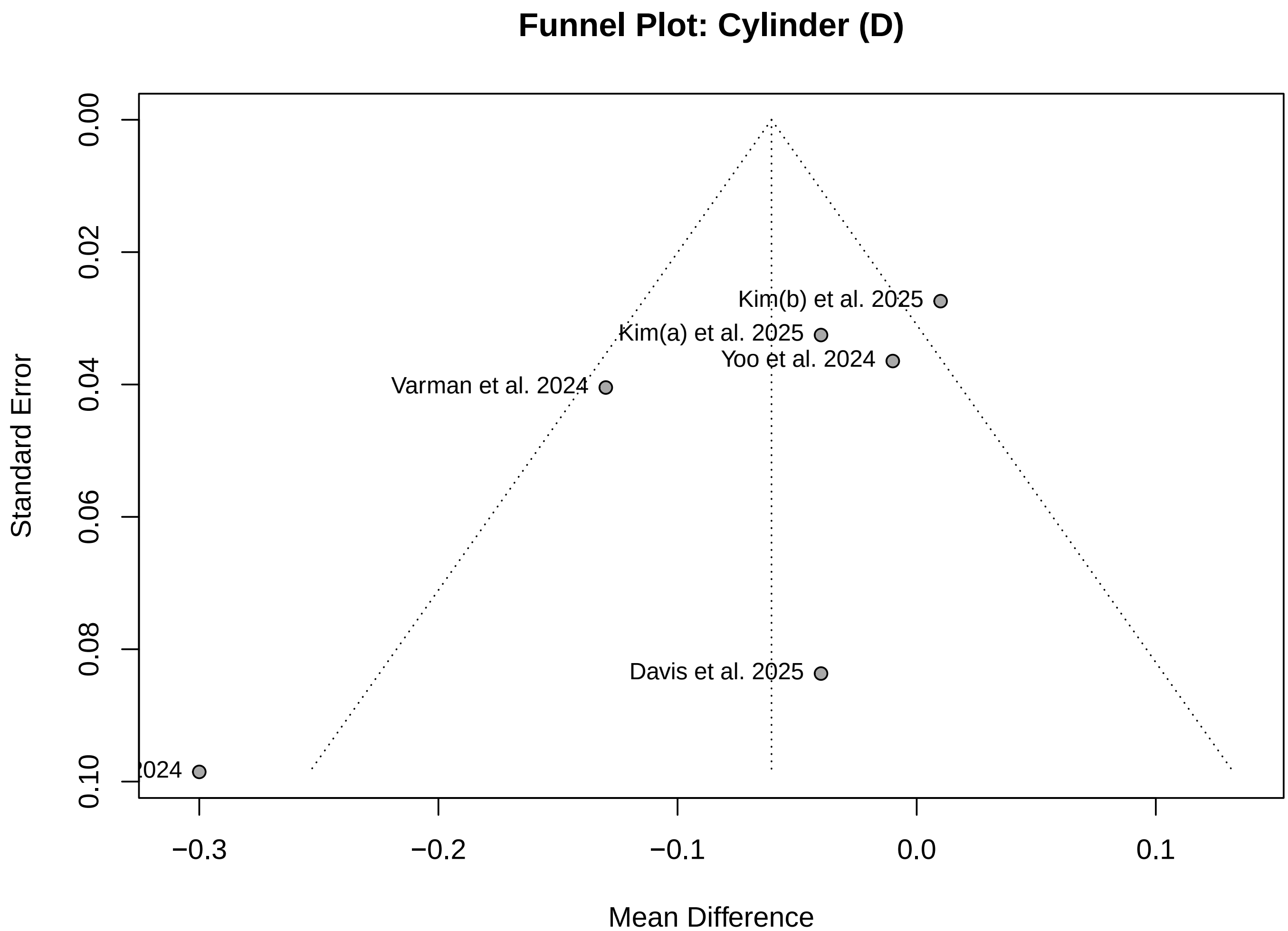

6 studies included. Egger's and Begg's tests require a minimum of 10 studies and were not performed.

6. SEQ (D)

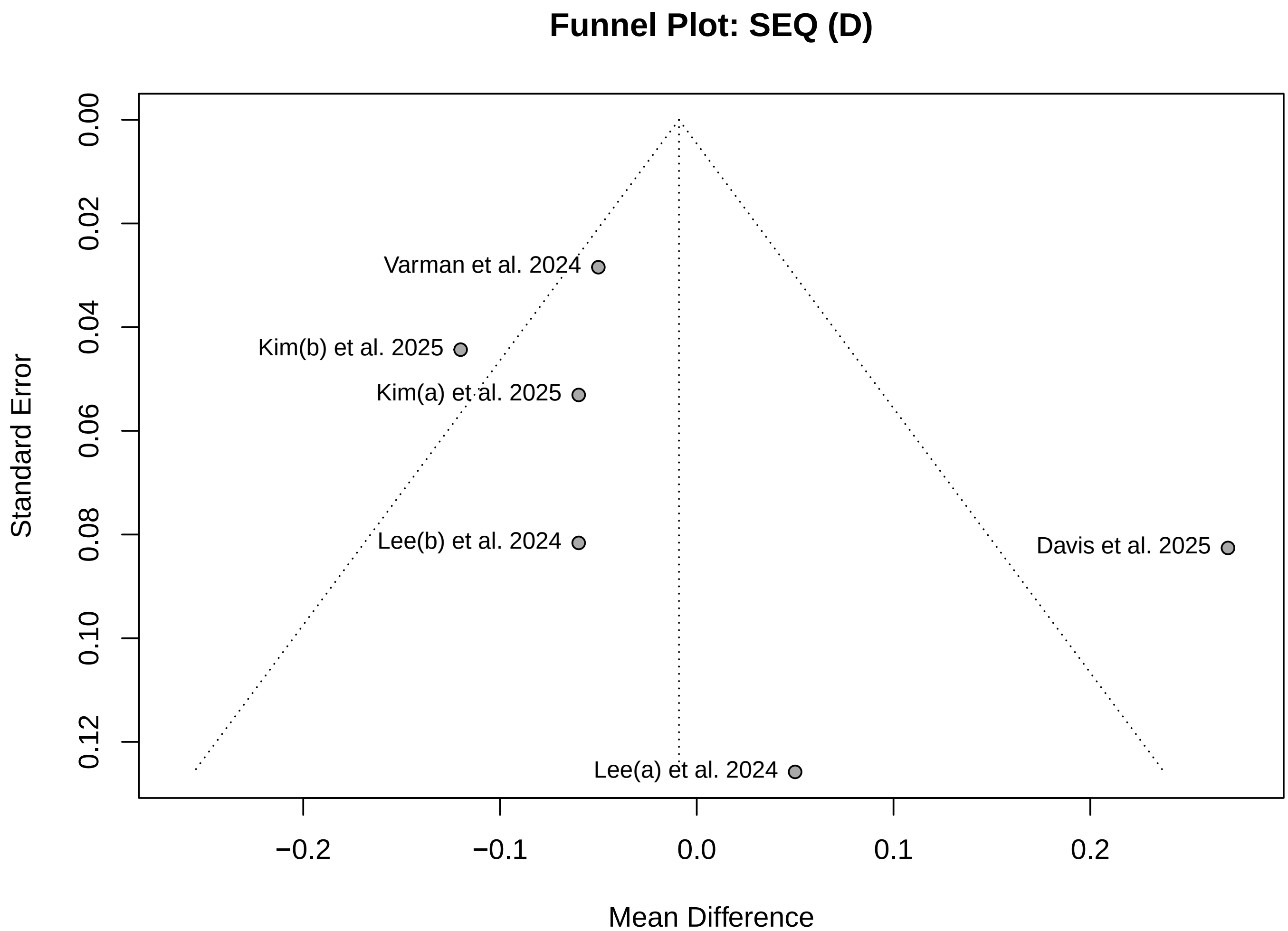

6 studies included. Egger's and Begg's tests require a minimum of 10 studies and were not performed.

7. Cyl <= 0.50D

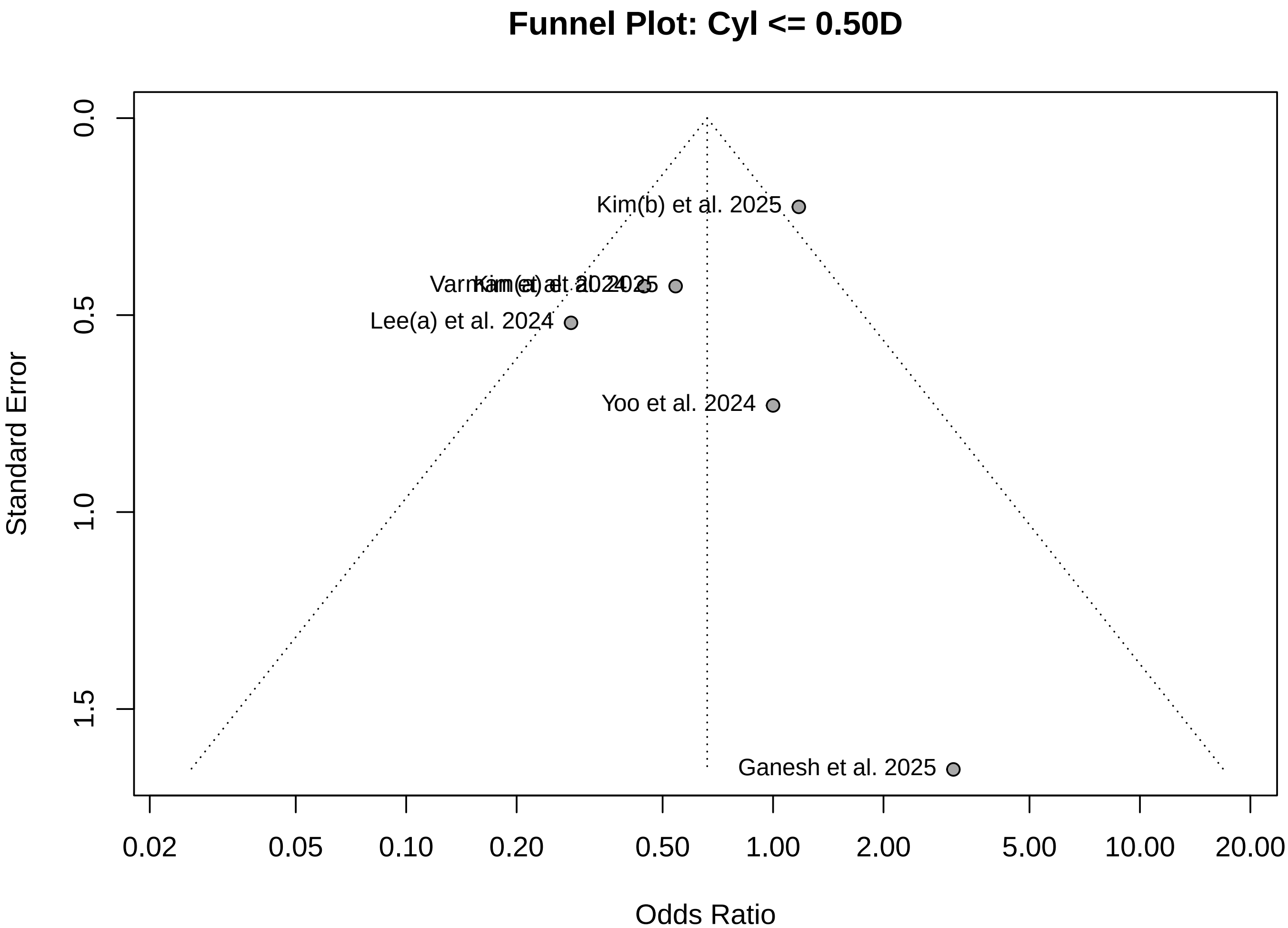

6 studies included. Egger's and Begg's tests require a minimum of 10 studies and were not performed.

8. SEQ within +/- 0.50D

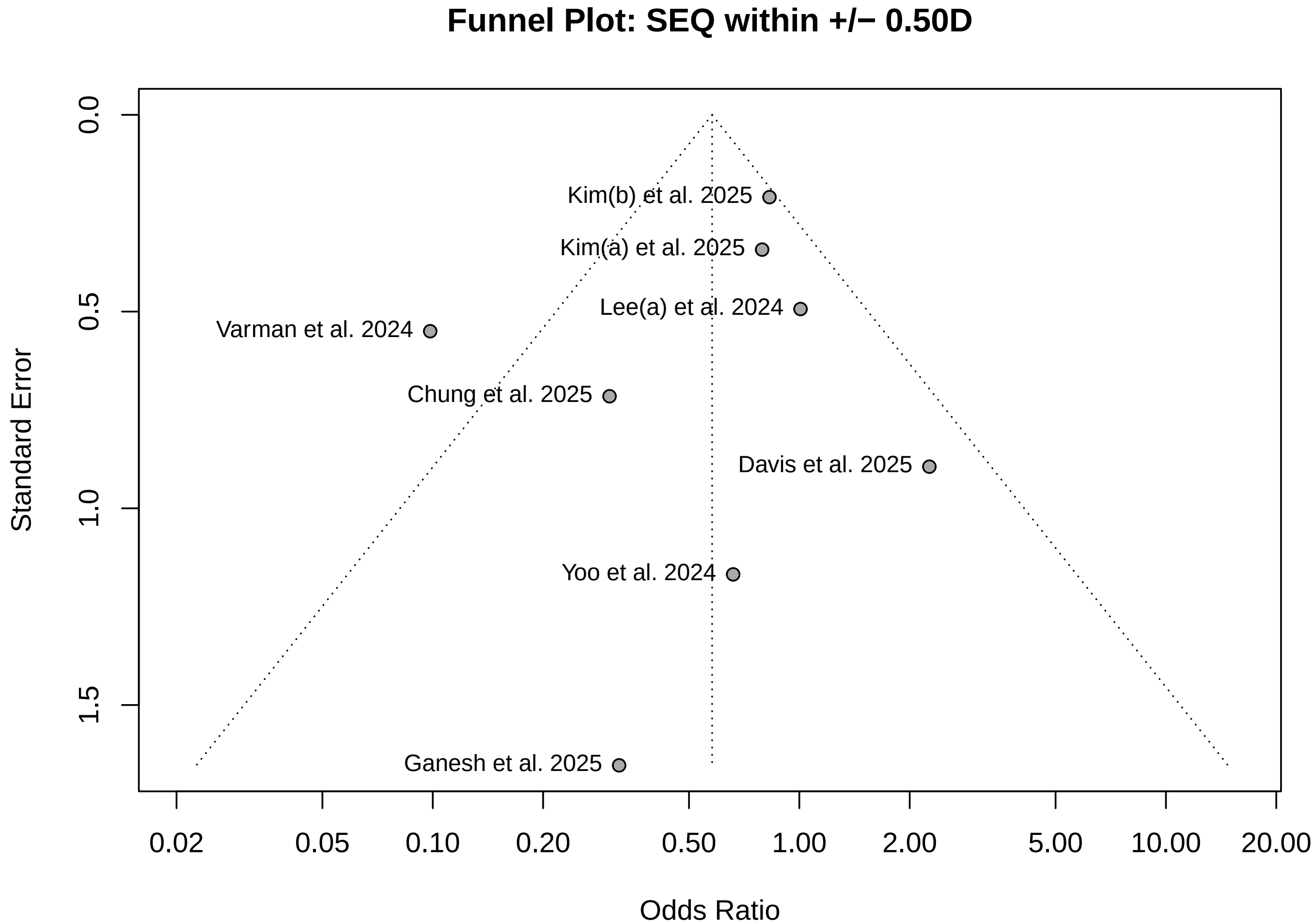

8 studies included. Egger's and Begg's tests require a minimum of 10 studies and were not performed.

9. TIA

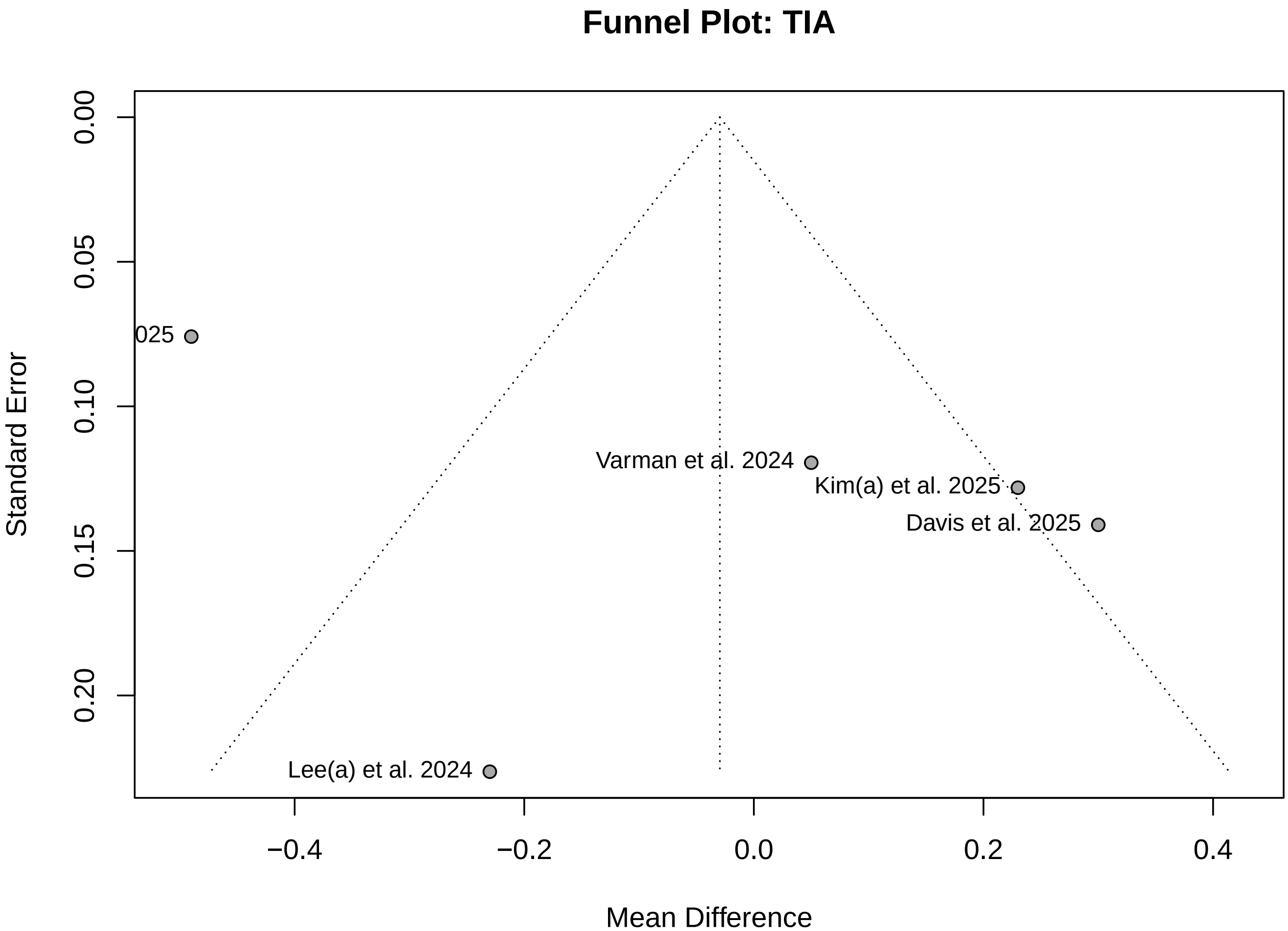

5 studies included. Egger's and Begg's tests require a minimum of 10 studies and were not performed.

10. SIA

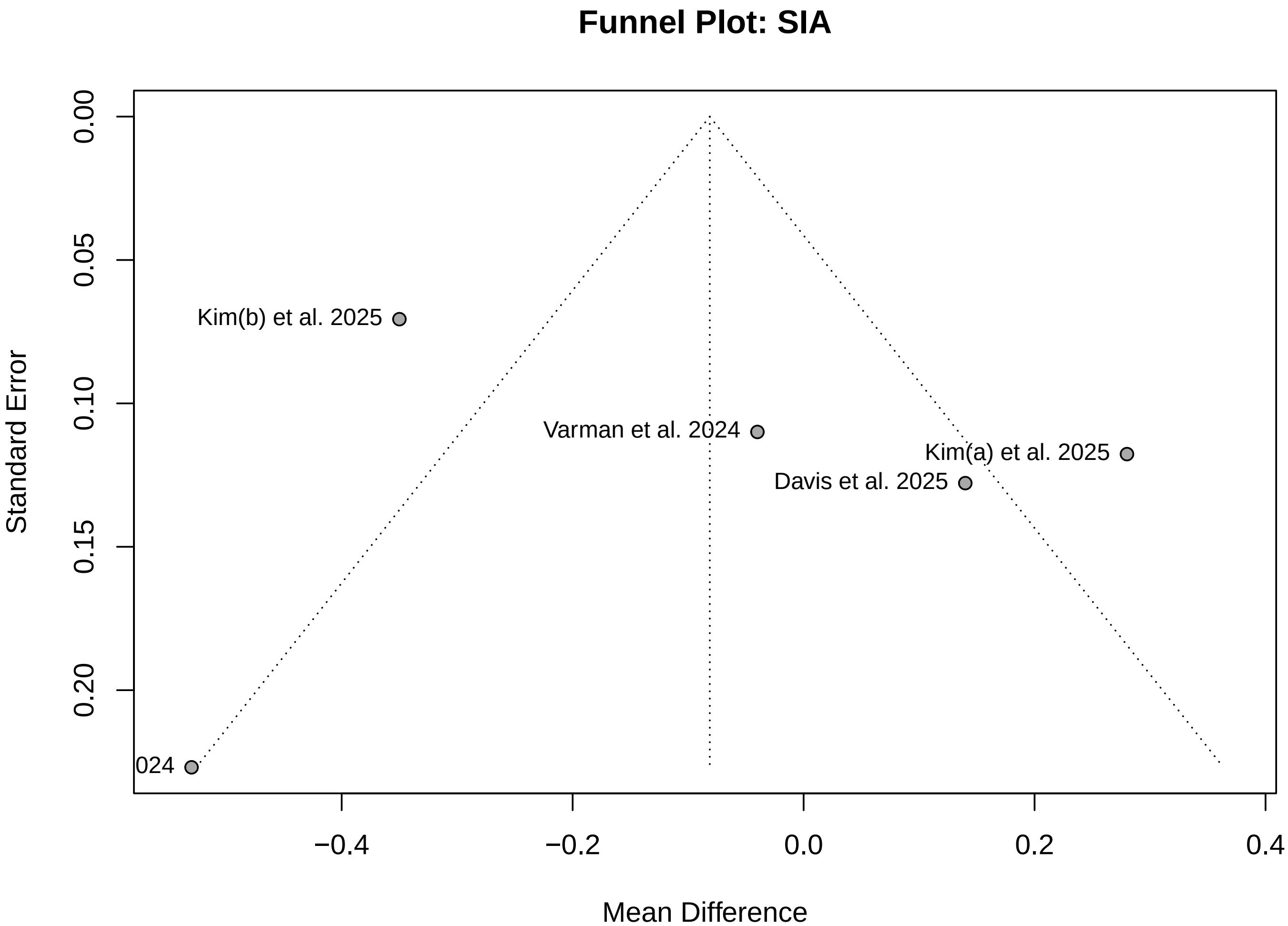

5 studies included. Egger's and Begg's tests require a minimum of 10 studies and were not performed.

11. AE

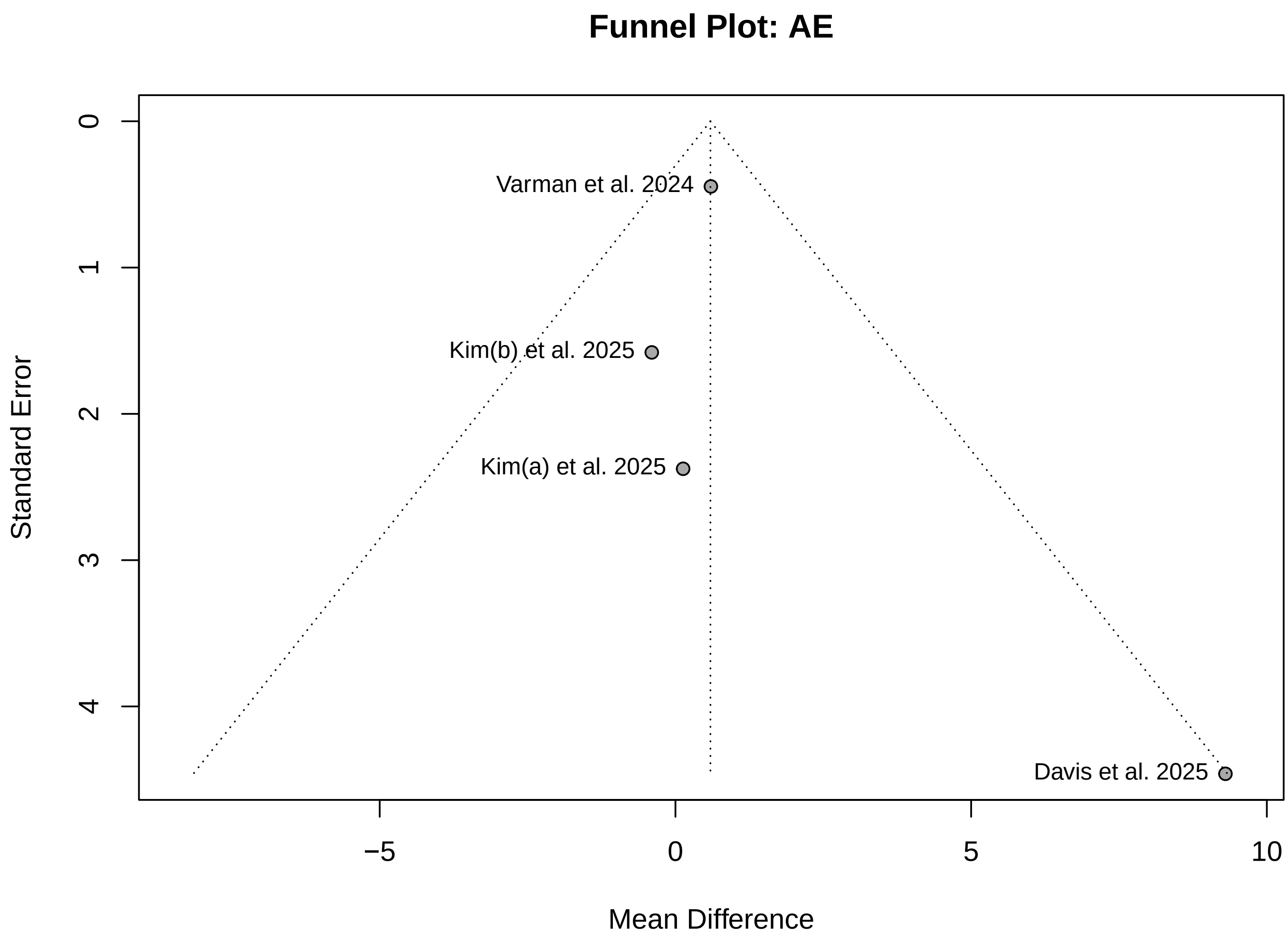

4 studies included. Egger's and Begg's tests require a minimum of 10 studies and were not performed.

12. |AE|

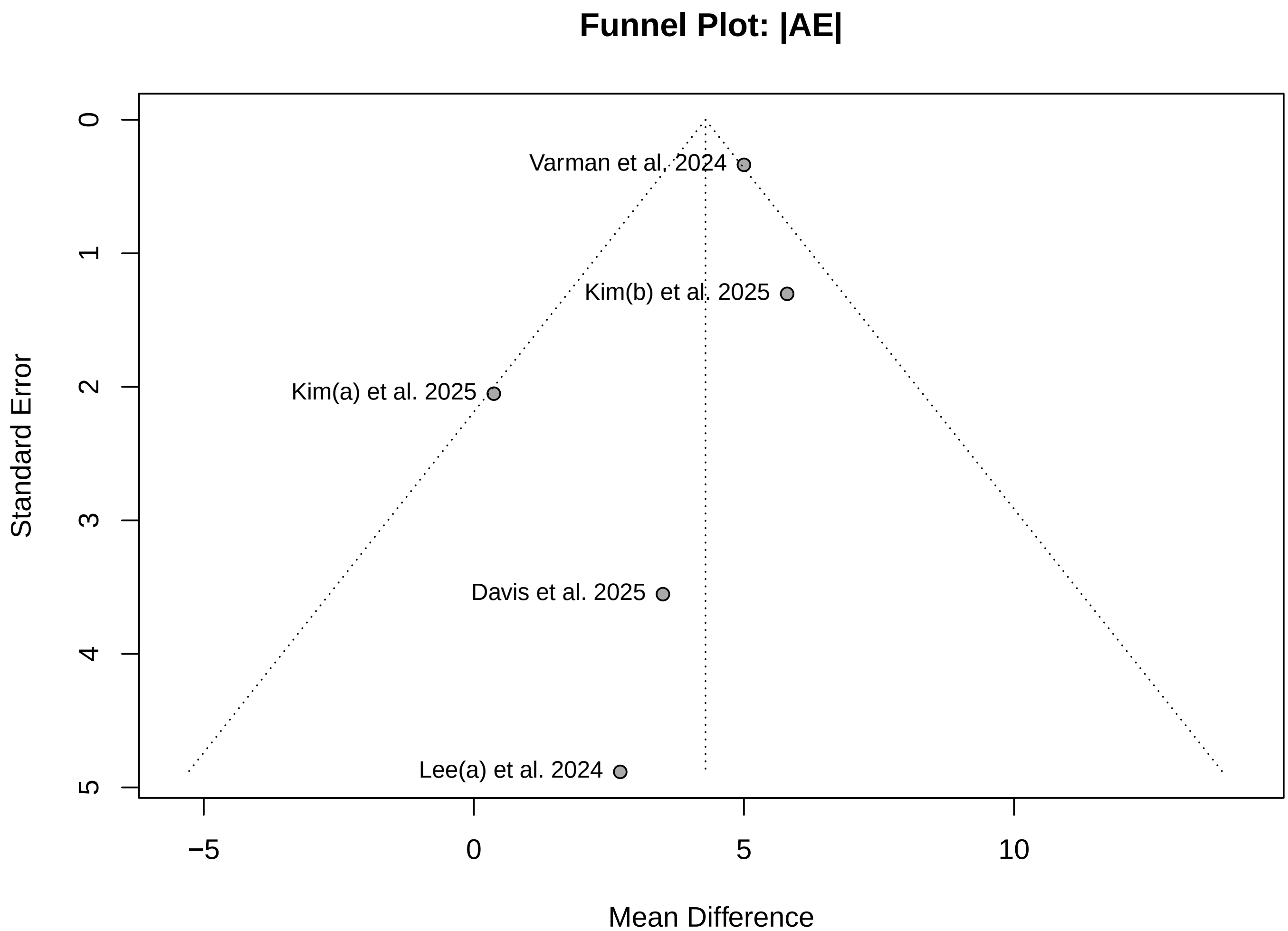

5 studies included. Egger's and Begg's tests require a minimum of 10 studies and were not performed.

13. Total HOAs

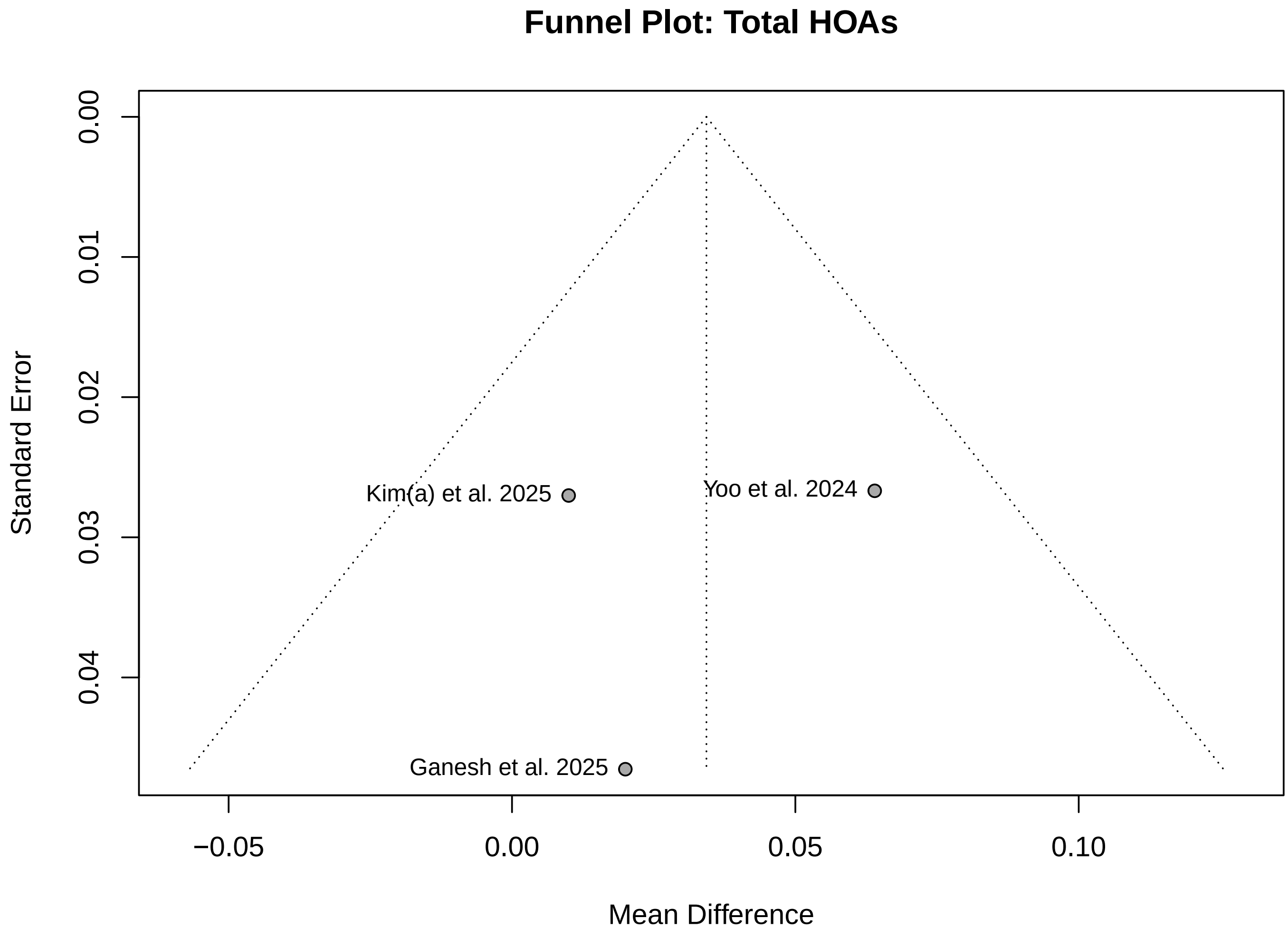

3 studies included. Egger's and Begg's tests require a minimum of 10 studies and were not performed.

14. Spherical Aberration

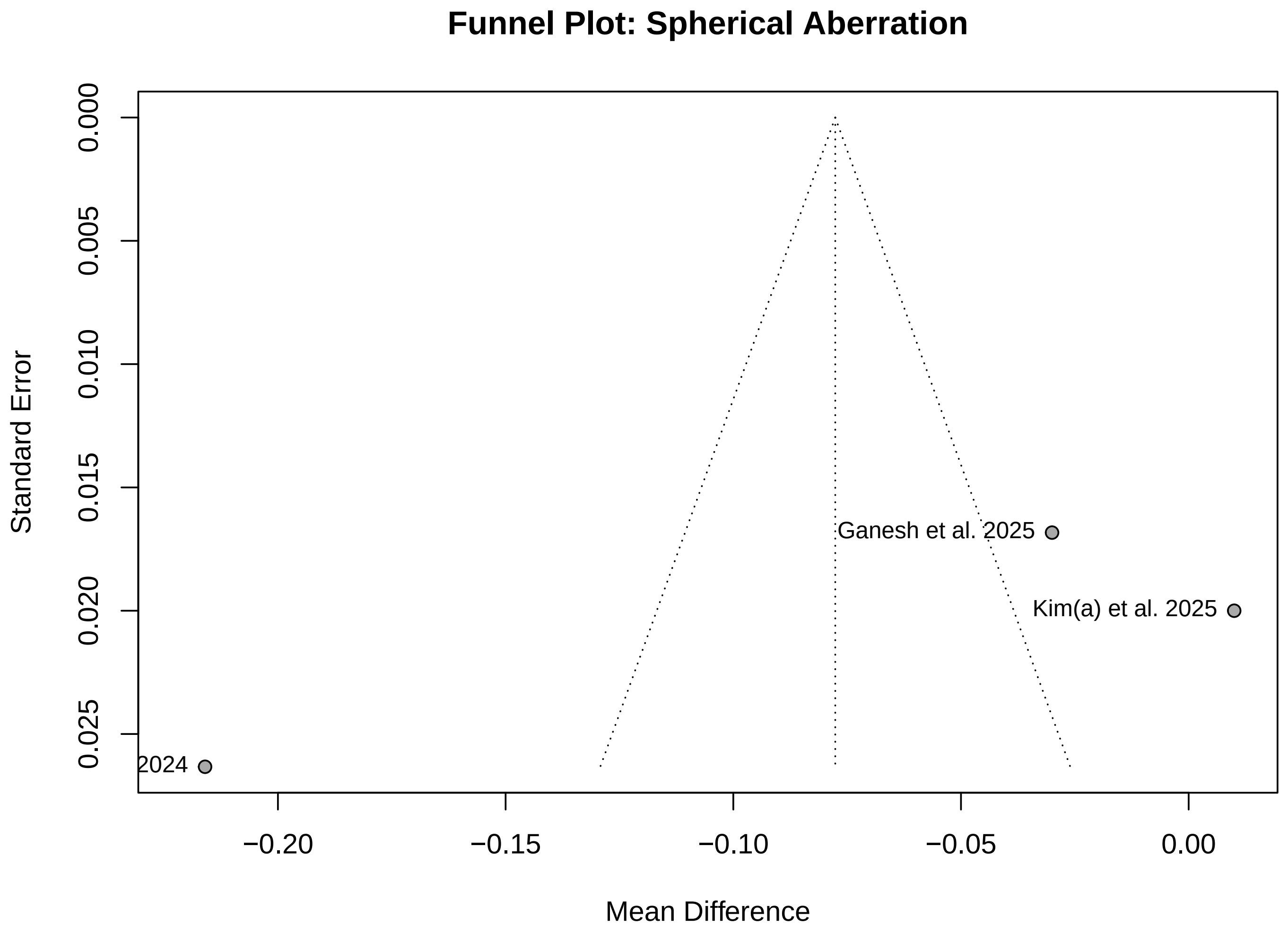

3 studies included. Egger's and Begg's tests require a minimum of 10 studies and were not performed.

15. Vertical Coma

Too few studies for publication bias assessment.

16. Horizontal Coma

Too few studies for publication bias assessment.

17. UDVA >= 20/40

Too few studies for publication bias assessment.

18. Lost 1 Line

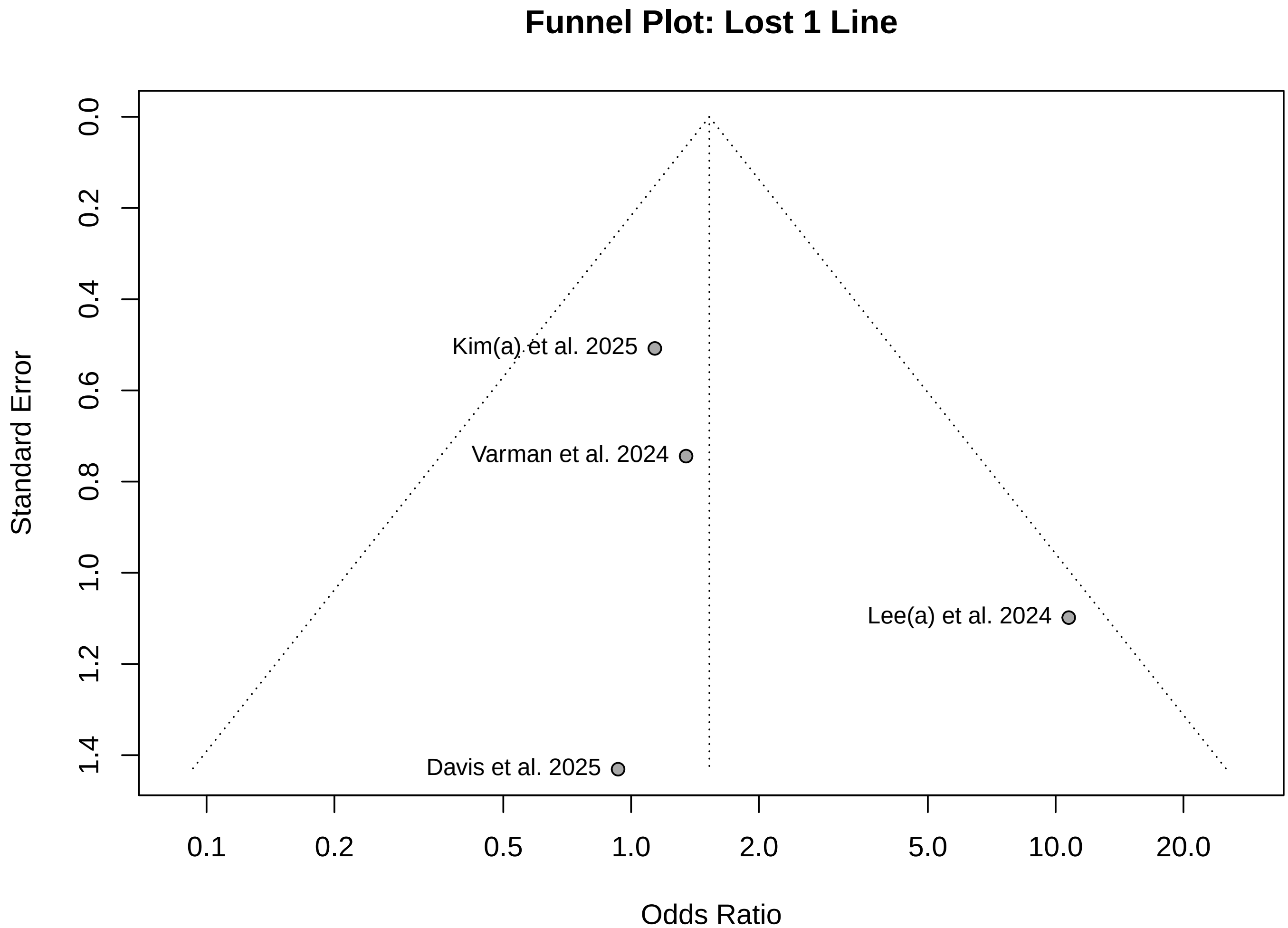

4 studies included. Egger's and Begg's tests require a minimum of 10 studies and were not performed.

19. No Change

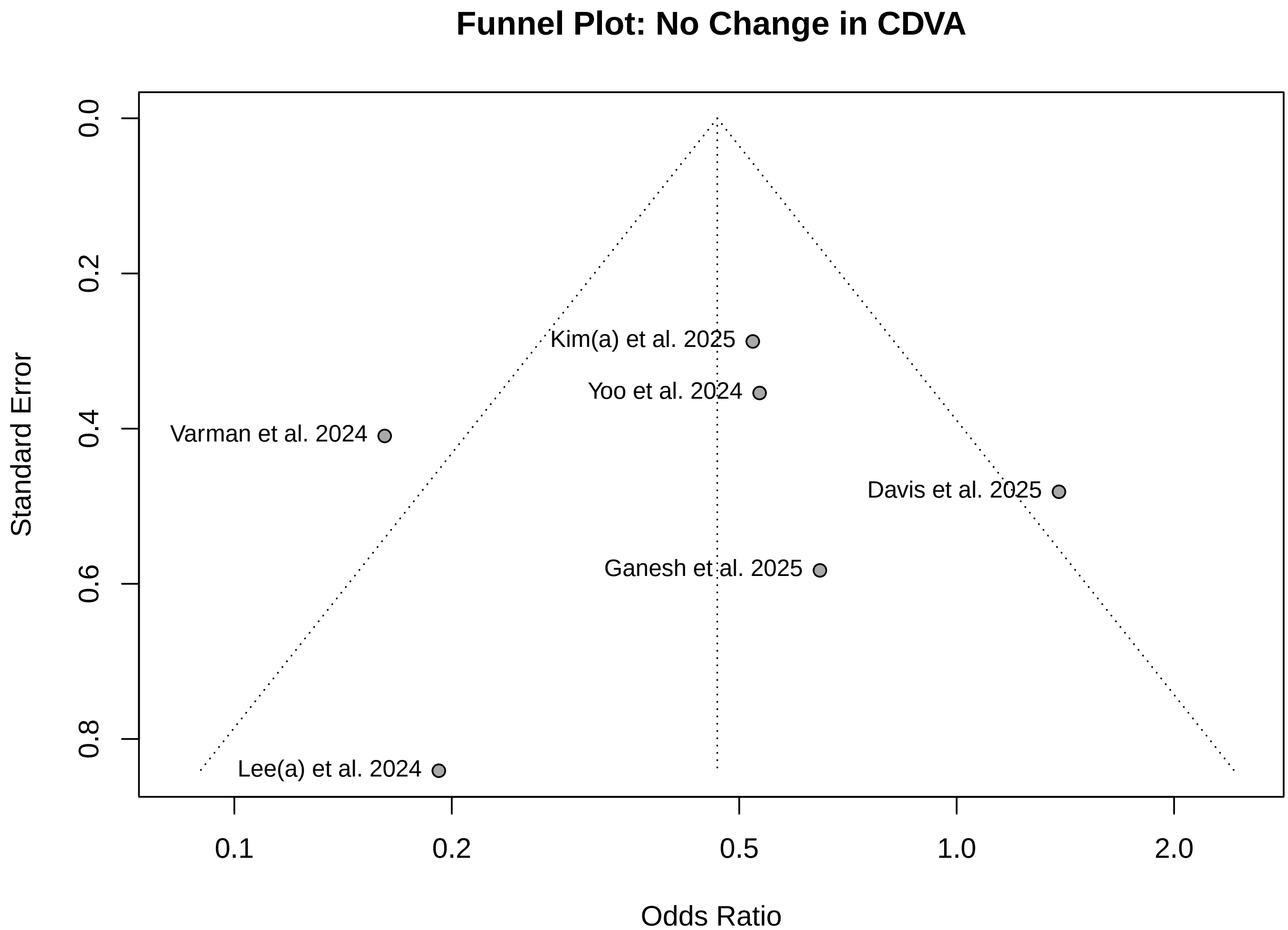

6 studies included. Egger's and Begg's tests require a minimum of 10 studies and were not performed.

20. Gain of 1 Line

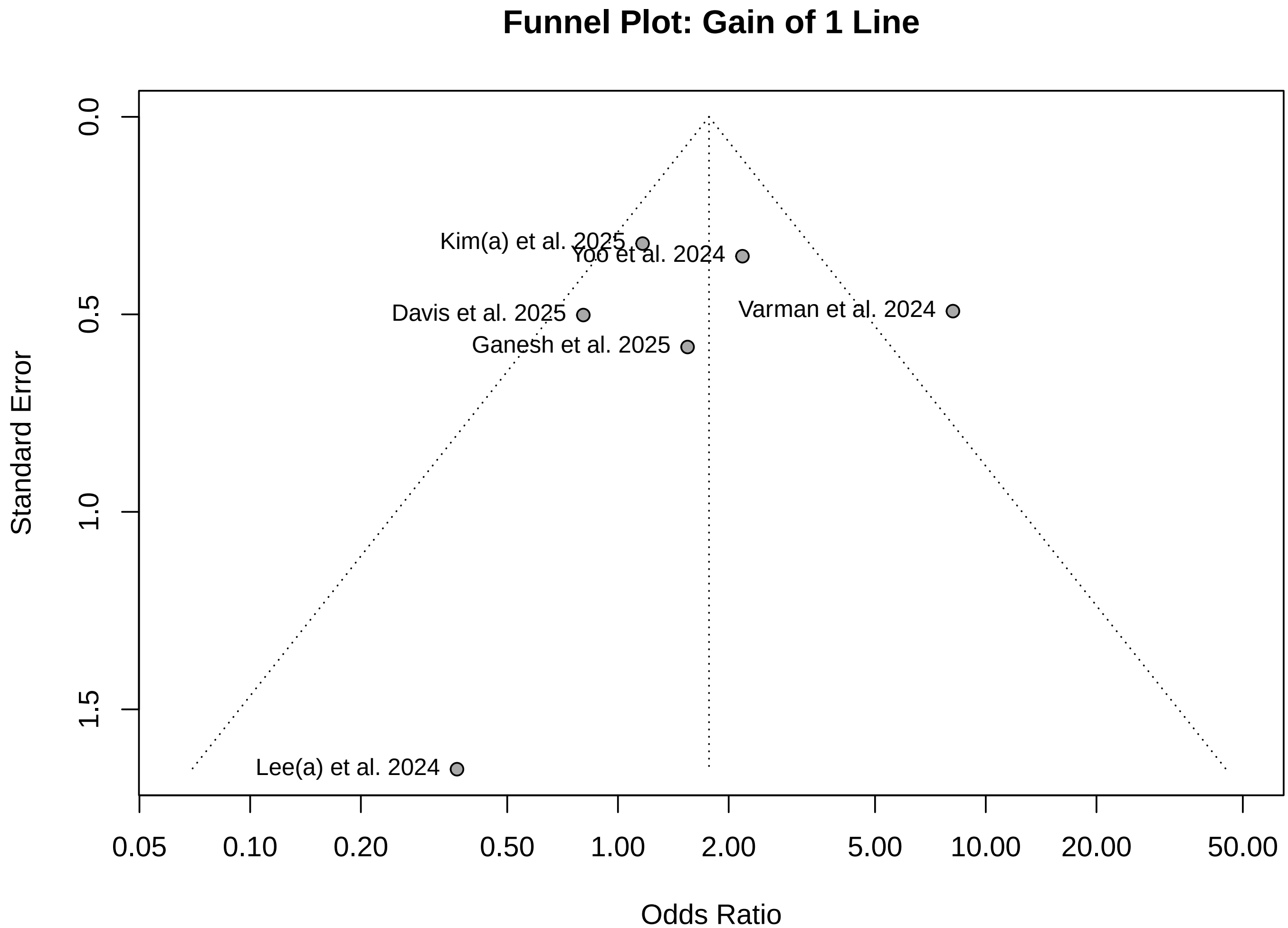

6 studies included. Egger's and Begg's tests require a minimum of 10 studies and were not performed.

21. Gain of 2 Lines

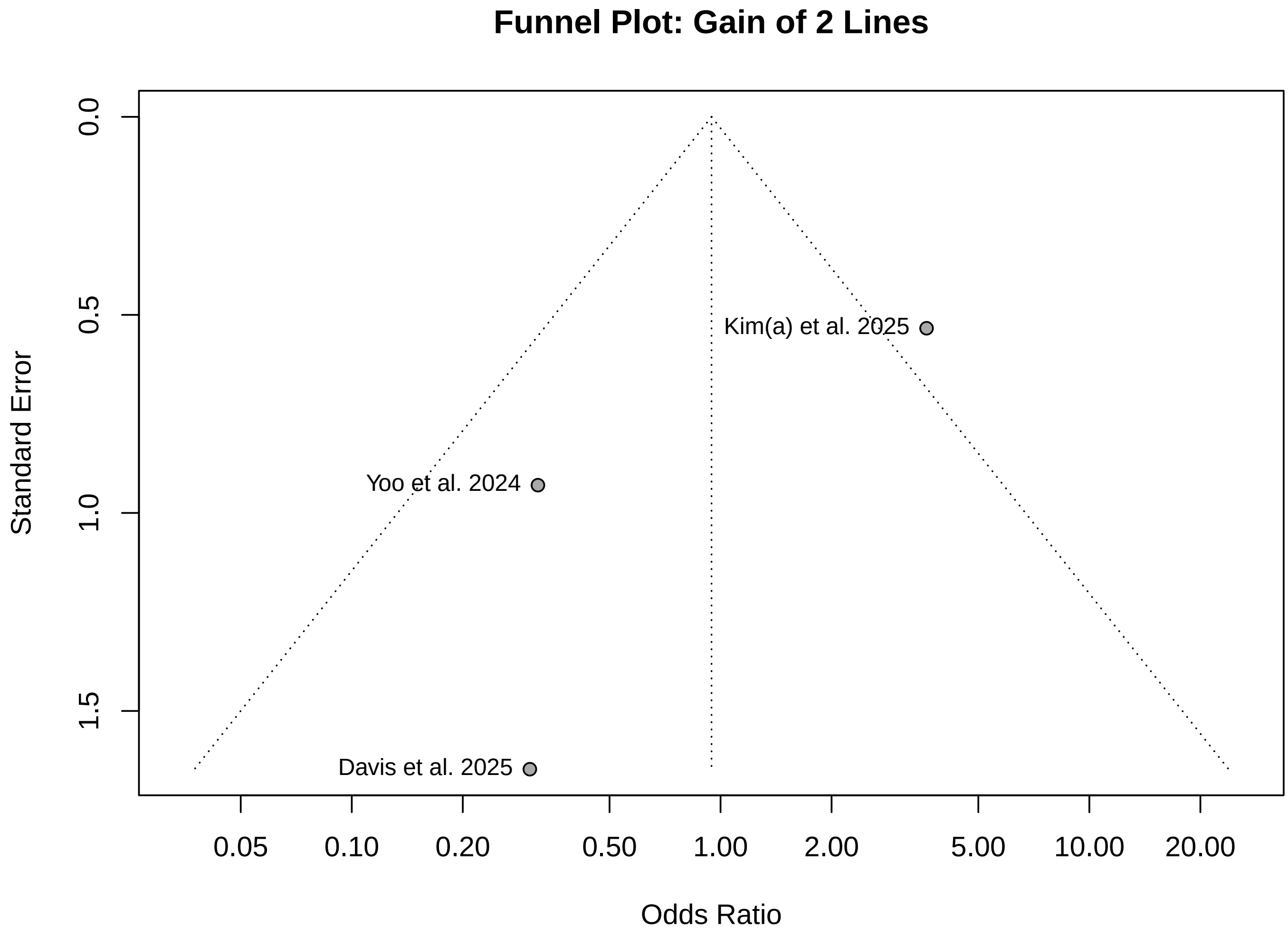

3 studies included. Egger's and Begg's tests require a minimum of 10 studies and were not performed.

22. Safety Index

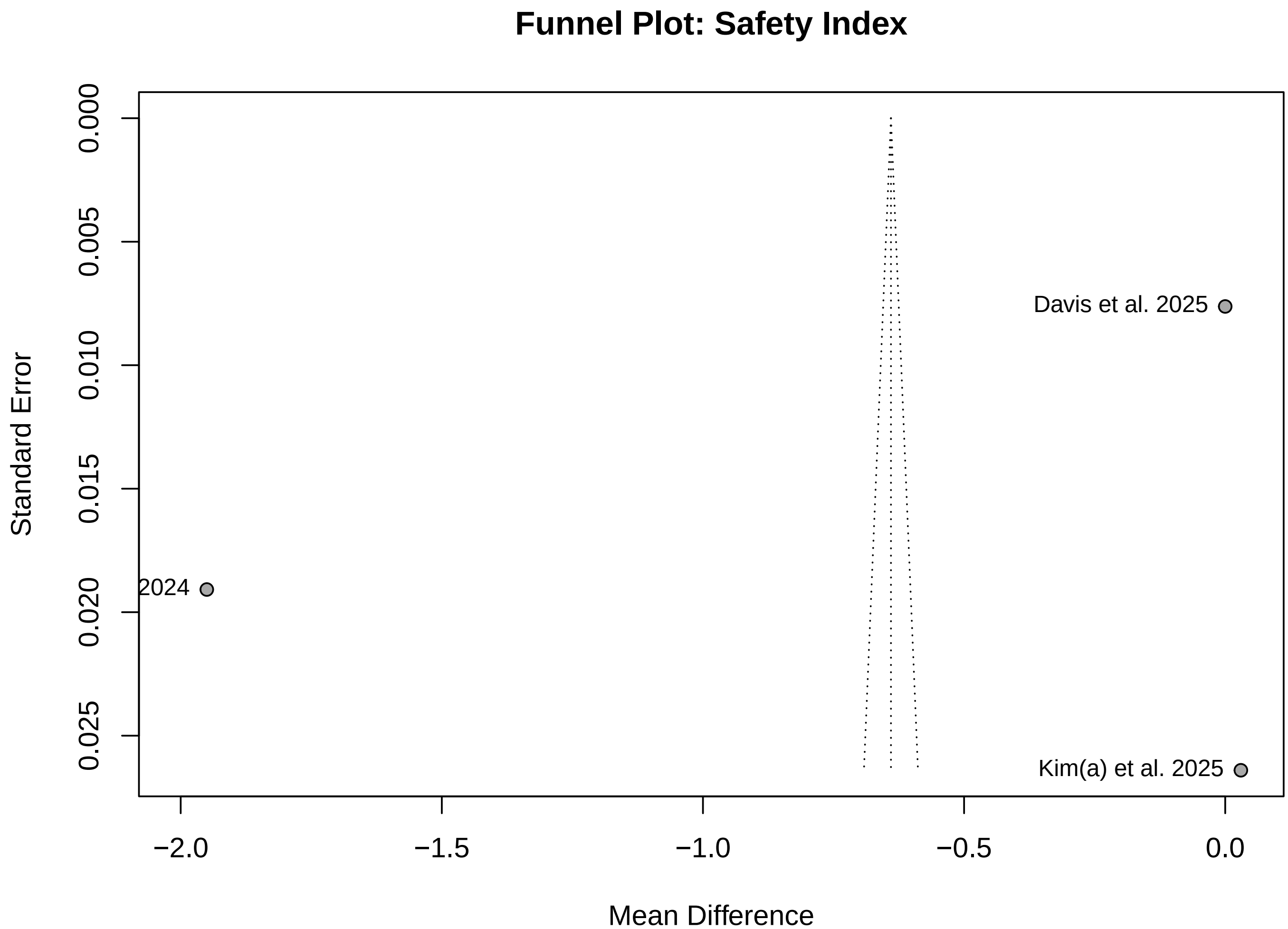

3 studies included. Egger's and Begg's tests require a minimum of 10 studies and were not performed.

23. Efficacy Index

Too few studies for publication bias assessment.
