# Supplementary material for: Comparison of outcomes after SMILE using VISUMAX 800 and VISUMAX 500 for correcting myopia and myopic astigmatism: a systematic review and meta-analysis
Source: Front Med (Lausanne). 2026 Oct 7;13:1899883. doi: 10.3389/fmed.2026.1899883 (PMC13683969; doi:10.3389/fmed.2026.1899883)
Supplement: Supplementary file 3 [file Datasheet3.doc]

Pubmed_search

1. "myopia"[MeSH Terms] OR "myopia"[Title/Abstract] OR "Myopic"[Title/Abstract] OR "Myopias"[Title/Abstract] OR "Nearsightedness"[Title/Abstract] OR "Shortsightedness"[Title/Abstract] OR "Shortsighted"[Title/Abstract] " astigmatism"[MeSH Terms] OR " astigmatism"[Title/Abstract] OR " refractive error"[Title/Abstract]
2. "corneal surgery, laser"[MeSH Terms] OR "corneal surgery laser"[Title/Abstract] OR "ReLEx"[Title/Abstract] OR "keratorefractive lenticule extraction"[Title/Abstract] OR "KLEx"[Title/Abstract] OR "femtosecond laser"[Title/Abstract]
3. "smile pro"[Title/Abstract] OR "visumax 800"[Title/Abstract] OR "second generation klex"[Title/Abstract] OR "visumax 800"[Title/Abstract] OR "visumax 500"[Title/Abstract] OR "VM500"[Title/Abstract] OR "small incision lenticule extraction"[Title/Abstract] OR "SMILE"[Title/Abstract]
4. "vision, ocular"[MeSH Terms] OR "vision ocular"[Title/Abstract] OR "vision"[Title/Abstract] OR "visual acuity"[Title/Abstract] OR "visual"[Title/Abstract] OR "aberrometry"[Title/Abstract] OR "spherical aberration"[Title/Abstract] OR "trefoil"[Title/Abstract] OR "coma"[Title/Abstract] OR "corneal higher order aberrations"[Title/Abstract] OR "HOAs"[Title/Abstract]

Cochrane_search

1. (myopia):ti,ab,kw OR (Myopic):ti,ab,kw OR (Myopias):ti,ab,kw OR (Nearsightedness):ti,ab,kw OR (Shortsightedness):ti,ab,kw OR (Shortsighted):ti,ab,kw OR (astigmatism):ti,ab,kw OR (refractive error):ti,ab,kw
2. (corneal surgery, laser):ti,ab,kw OR (ReLEx):ti,ab,kw OR (keratorefractive lenticule extraction):ti,ab,kw OR (KLEx):ti,ab,kw OR (femtosecond laser):ti,ab,kw
3. (Smile Pro):ti,ab,kw OR (VisuMax 800):ti,ab,kw OR (second generation KLEx):ti,ab,kw OR (VM800):ti,ab,kw OR (VisuMax 500):ti,ab,kw OR (VM500):ti,ab,kw OR (small incision lenticule extraction):ti,ab,kw OR (SMILE):ti,ab,kw
4. (vision, ocular):ti,ab,kw OR (vision):ti,ab,kw OR (visual acuity):ti,ab,kw OR (visual):ti,ab,kw OR (aberrometry):ti,ab,kw OR (spherical aberration):ti,ab,kw OR (trefoil):ti,ab,kw OR (coma):ti,ab,kw OR (corneal higher-order aberrations):ti,ab,kw OR (HOAs):ti,ab,kw

Embase _search

1. 1.myopia:ab,ti OR myopic:ab,ti OR myopias:ab,ti OR nearsightedness:ab,ti OR shortsightedness:ab,ti OR shortsighted:ab,ti OR astigmatism:ab,ti OR 'refractive error':ab,ti
2. 'photorefractive keratectomy':ab,ti OR 'corneal surgery, laser':ab,ti OR ReLEx:ab,ti OR 'keratorefractive lenticule extraction':ab,ti OR 'femtosecond laser':ab,ti OR KLEx:ab,ti
3. VM500:ab,ti OR VM800:ab,ti OR SMILE:ab,ti OR 'Smile Pro':ab,ti OR 'VisuMax 800':ab,ti OR 'second generation KLEx':ab,ti OR 'VisuMax 500':ab,ti OR 'small incision lenticule extraction':ab,ti
4. vision:ab,ti OR visual:ab,ti OR aberrometry:ab,ti OR 'vision, ocular':ab,ti OR 'visual acuity':ab,ti OR 'spherical aberration':ab,ti OR 'corneal higher-order aberrations':ab,ti trefoil:ab,ti OR coma:ab,ti OR HOAs:ab,ti
